# Supplementary material for: Organization of feedback projections to mouse primary visual cortex
Source: iScience. 2021 Apr 17;24(5):102450. doi: 10.1016/j.isci.2021.102450 (PMC8169797; doi:10.1016/j.isci.2021.102450)
Supplement: Document S1. Figures S1–S7 and Tables S1 and S3–S6 [file mmc1.pdf]

iScience, Volume *24*

Supplemental information

Organization of feedback projections to mouse primary visual cortex

Mai M. Morimoto, Emi Uchishiba, and Aman B. Saleem

Supplementary Tables

Table S1 (related to Figures 2, 3): Area name abbreviations used in the study. Most follow Allen CCF (v3) nomenclature (Wang et al., 2020). Exceptions are indicated in italic, and corresponding Allen CCF (v3) nomenclature shown in brackets where existing.

V1 (VISp)	Primary visual area
AUD	Auditory areas
MO	Somatomotor areas
SS	Somatosensory areas
RSP	Retrosplenial area
RSPagl	Retrosplenial area, lateral agranular part
RSPd	Retrosplenial area, dorsal part
RSPv	Retrosplenial area, ventral part
ACA	Anterior cingulate area
TEa	Temporal association areas
ECT	Ectorhinal area
MEC (ENTm)	Entorhinal area, medial part, dorsal zone
LEC (ENTl)	Entorhinal area, lateral part
CLA	Claustrum
SUBcom	Subicular complex
PAR	Parasubiculum
POST	Postsubiculum
PRE	Presubiculum
PM (VISpm)	Posteromedial visual area
AM (VISal)	Anteromedial visual area
A (VISa)	Anterior area
RL (VISrl)	Rostrolateral visual area
AL (VISal)	Anterolateral visual area
LM (VISl)	Lateromedial visual area
LI (VISli)	Laterointermediate area
POR (VISpor)	Postrhinal area
PL (VISpl)	Posterolateral visual area

Table S3 (related to Figure 3): Number of cells detected across all brain areas.

Area	% cell count			Absolute cell count	
	Mean	SEM	Median	Total cells counted	from ‘n = ’ injections
AUD	7.465	0.679	6.615	5895	16
MO	1.606	0.224	1.645	1464	16
SS	3.063	0.402	2.652	2543	16
ACA	3.332	0.433	3.172	2777	16
ECT	0.669	0.098	0.601	537	16
LEC	0.545	0.092	0.457	476	16
MEC	1.750	0.227	1.804	1558	16
RSPagl	6.484	0.954	4.975	5144	16
RSPd	8.419	0.794	7.906	6714	16
RSPv	4.107	0.534	3.588	3255	16
TEa	5.279	0.437	5.284	4220	16
CLA	2.270	0.331	2.320	2063	16
SUBcom	1.197	0.139	1.171	990	16
PM	7.837	0.648	7.988	6991	21
AM	6.467	1.066	5.163	5317	21
A	3.039	0.712	1.908	2531	21
RL	4.965	1.245	2.780	3348	21
AL	6.358	0.521	5.940	5698	21
LM	15.940	1.748	14.581	14095	21
LI	5.575	0.440	5.458	4814	21
POR	8.830	0.787	8.573	7470	21
PL	7.401	0.865	6.389	5898	21

Table S4 (related to Figure 5): Measure of topography across areas. Percentage of voxels per area with significant retinotopic projection selectivity for V1.

		azimuth		elevation	
**Area**	**Voxel count**	**p<0.1 (%)**	**p<0.05 (%)**	**p<0.1 (%)**	**p<0.05 (%)**
AUD	1904	9.30	4.31	6.41	2.15
MO	738	7.45	1.90	12.06	2.57
SS	1456	9.00	1.17	14.42	1.58
ACA	870	6.44	1.03	9.89	2.64
CLA	242	13.22	2.48	6.20	3.72
TEa	1004	10.96	3.09	10.26	4.78
RSP	3262	16.62	4.69	12.05	3.83
ECT	281	6.41	1.07	7.83	0.36
SUBcom	292	13.70	2.40	5.82	1.71
LEC	335	5.97	0.90	1.19	0.60
MEC	556	4.68	0.90	3.78	0.90
PM	633	17.22	6.48	12.01	5.85
AM	453	13.47	7.51	10.82	7.06
A	595	13.95	2.86	10.59	7.23
RL	476	17.44	2.31	8.40	3.57
AL	463	14.25	6.91	13.17	7.78
LM	772	11.40	3.50	14.25	5.96
LI	335	10.15	3.88	10.15	4.18
POR	555	14.23	9.73	11.71	6.85
PL	399	11.78	5.76	10.53	6.27

Table S5 (related to Figure 6): Measure of bias in projections to V1. Correlation coefficient and p-values for cell count to azimuth and elevation retinotopy correlation. ($p<0.05$ in red)

**Areas**	**Azimuth correlation r (p)**	**Elevation correlation r (p)**
AUD	0.0188 (0.9448)	-0.3029 (0.2541)
MO	0.1845 (0.4939)	-0.0051 (0.9850)
SS	-0.0750 (0.7826)	0.1935 (0.4727)
ACA	0.1840 (0.4952)	-0.2517 (0.3470)
CLA	0.3484 (0.2031)	-0.2796 (0.3128)
Tea	-0.0101 (0.9705)	-0.4088 (0.1159)
RSPagl	0.7372 (0.0011)	-0.4618 (0.0717)
RSPd	0.5132 (0.0420)	-0.2747 (0.3032)
RSPv	-0.0275 (0.9196)	-0.2157 (0.4223)
ECT	0.4262 (0.0997)	-0.6089 (0.0123)
PRE	-0.1667 (0.5373)	0.4901 (0.0539)
POST	0.5202 (0.0389)	-0.1317 (0.6268)
PAR	-0.0269 (0.9242)	-0.3958 (0.1442)
LEC	0.0405 (0.8860)	0.0260 (0.9266)
MEC	-0.0076 (0.9785)	-0.0858 (0.7610)
PM	0.7284 (0.0002)	-0.6241 (0.0025)
AM	0.5138 (0.0172)	-0.3305 (0.1434)
A	0.3687 (0.1001)	-0.0384 (0.8687)
RL	-0.5545 (0.0091)	0.3757 (0.0933)
AL	-0.1493 (0.5183)	-0.0395 (0.8649)
LM	-0.3024 (0.1827)	0.4734 (0.0302)
LI	0.5161 (0.0166)	-0.5227 (0.0151)
POR	-0.4690 (0.0320)	-0.0161 (0.9448)
PL	-0.4902 (0.0241)	0.2231 (0.3310)

Table S6 (related to Figure 6): Layer distribution of cells in the source area projecting to V1, measured as the percentage of supragranular labelled neurons (%SLN).

Higher Visual Areas		Non-Visual Areas	
	**%SLN**		**%SLN**
**RL**	9.60	**MEC**	0.06
**POR**	10.00	**LEC**	3.51
**PL**	11.18	**ACA**	4.37
**A**	14.53	**MO**	6.42
**LI**	14.49	**RSPv**	8.36
**AM**	17.68	**TEa**	8.71
**AL**	18.74	**ECT**	10.02
**LM**	20.52	**SS**	12.78
**PM**	24.39	**AUD**	14.40
		**RSPd**	15.03
		**RSPagl**	22.74

Supplementary Figures

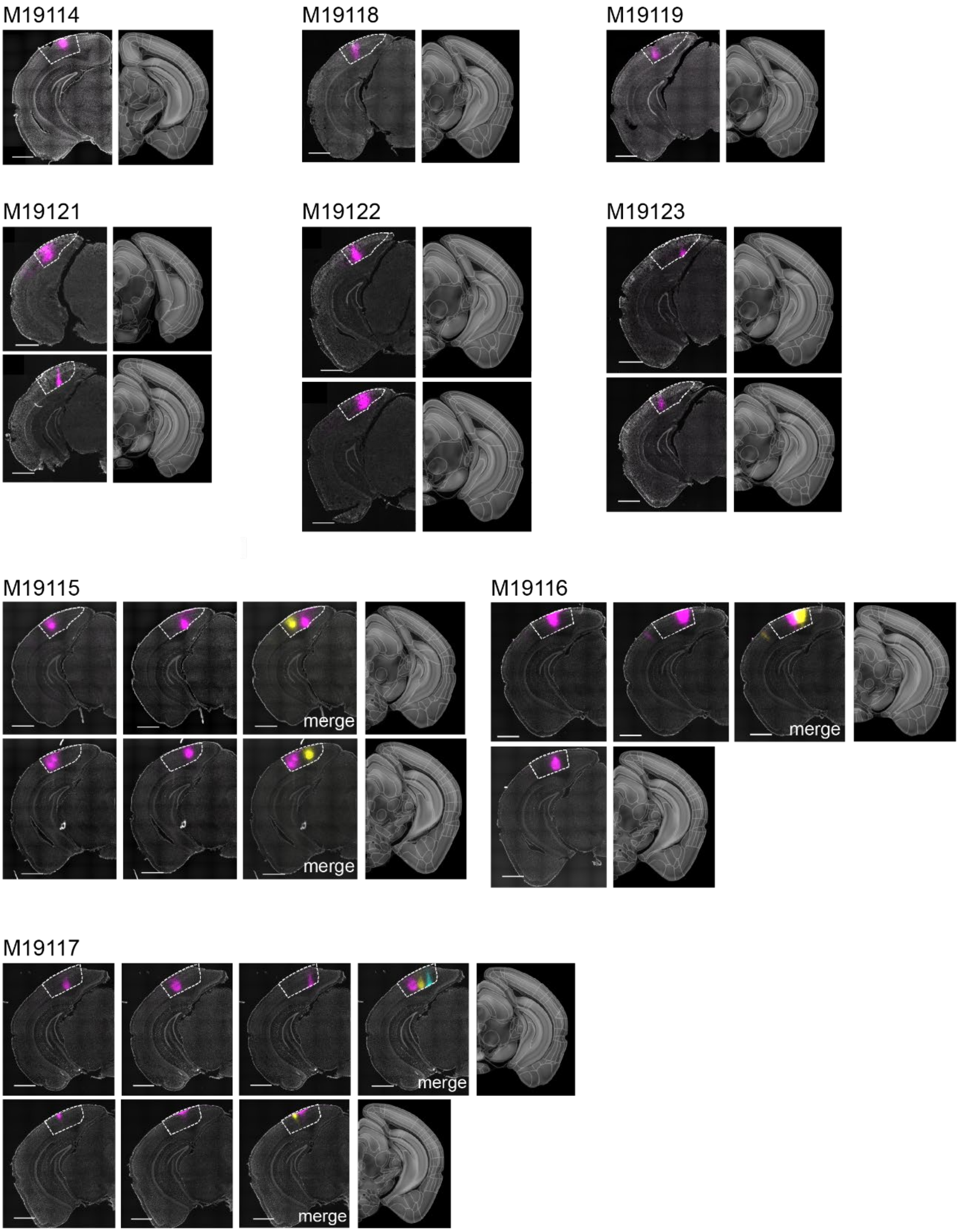

Figure S1 (related to Figure 1): CTB injection sites used in the study (9 animals, 21 injections). Fluorescent signal from each injection is shown in magenta. In 'merge' images for multiple injections within the same hemisphere, additional injections are shown in yellow or cyan. For each brain image (left hemisphere image), the ARA image used for alignment is shown on the same row (right hemisphere image). The brain images are pre-morphing, before alignment to the reference image. Border of V1 is indicated in white dotted lines according to this ARA image. Scale bars = 1mm. Further details of injections in methods and Table 1.

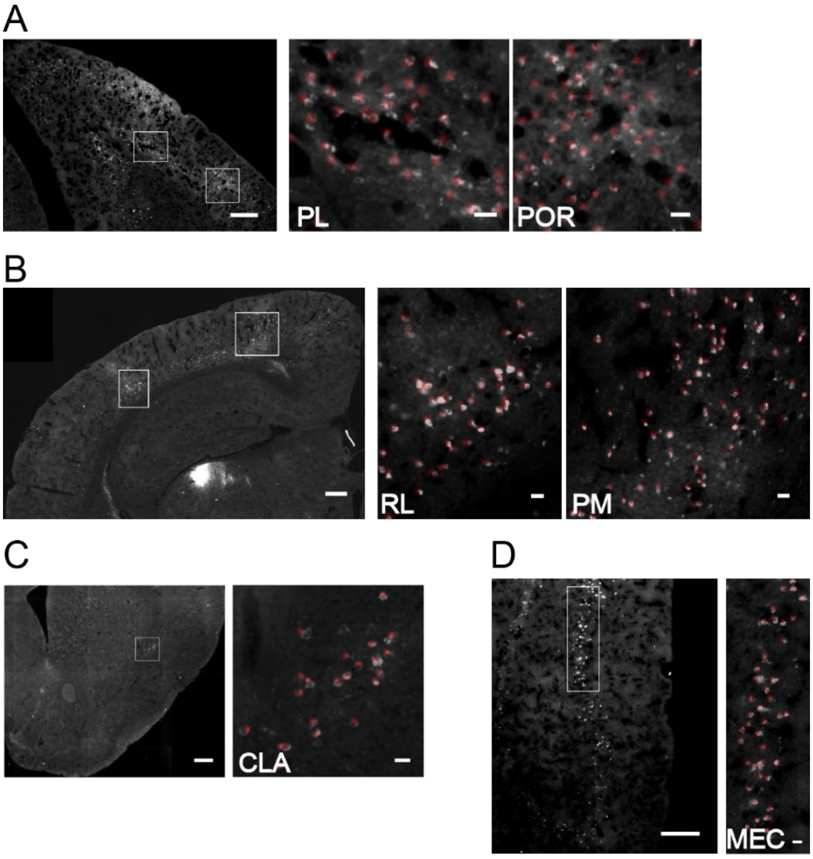

Figure S2 (related to Figure 1): Efficacy of cell-detection. **A-D)** Cell detection through our software pipeline. Red regions indicate detected cells. Right panels correspond to white boxed regions on the left panel. Scale bars: left panel = 100µm, right panel = 10µm.

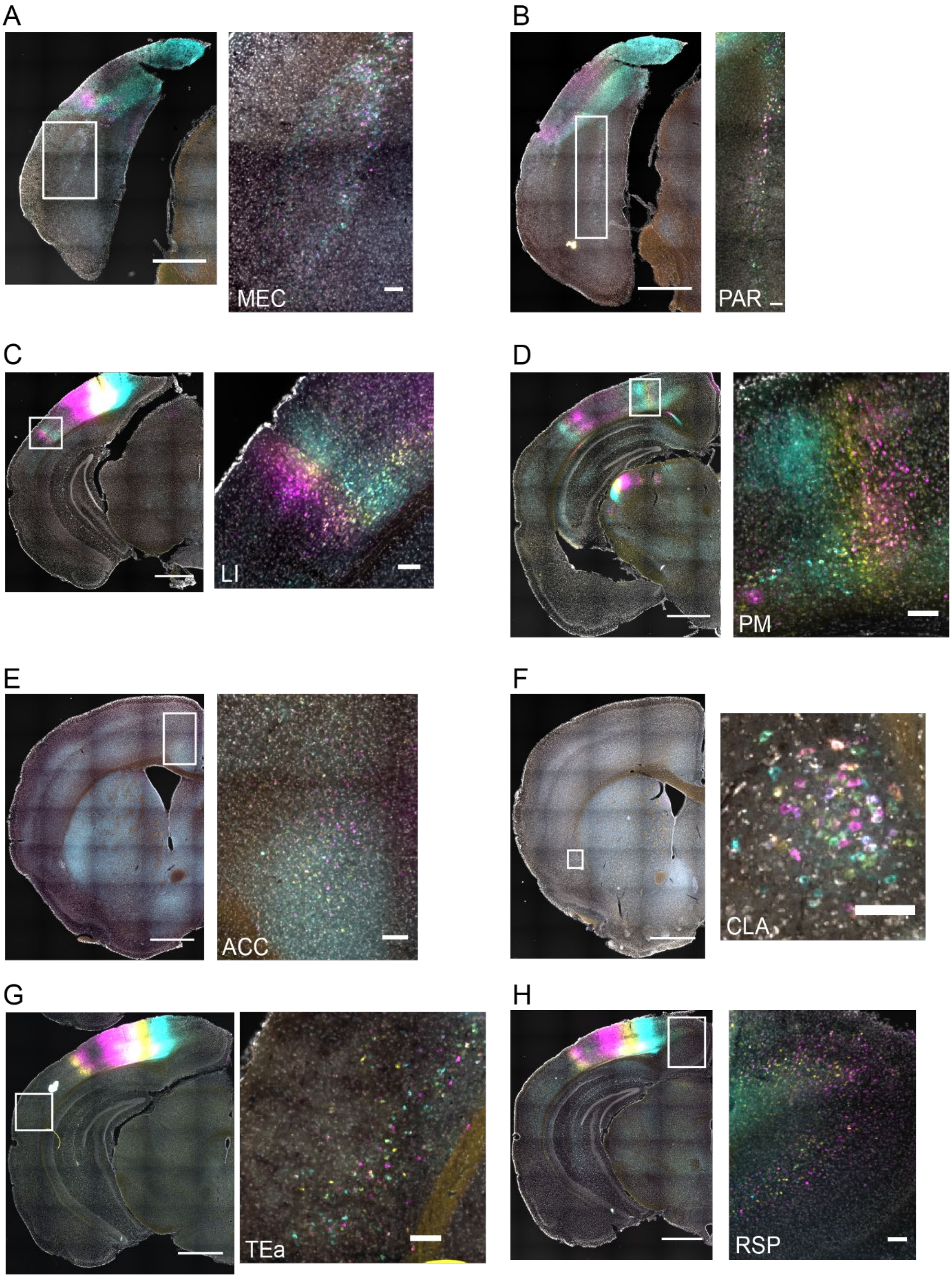

Figure S3 (related to Figure 2): Retrogradely labelled cells detected in various brain areas. **A-H)** Example areas showing retrogradely labelled cells. Right panels correspond to white boxed regions on the left panel. Scale bars: left panel = 1 mm, right panel = 100 µm.

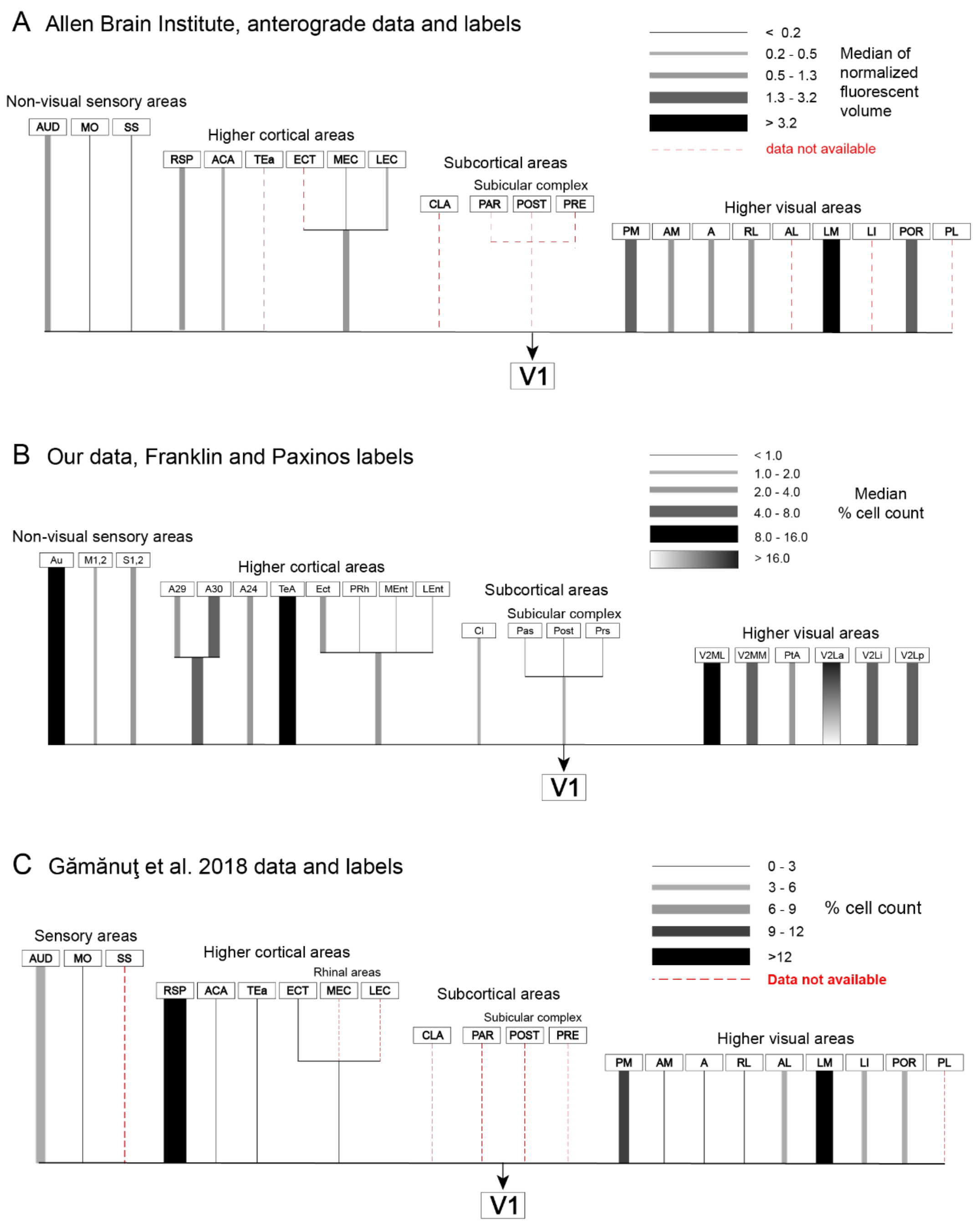

Figure S4 (related to Figure 3): Proportion of projections to V1 based on different atlases, and data. **A)** Estimated projection to V1 from brain wide anterograde injection data from the Allen Brain Institute. Injections to these areas were curated for specificity and projection to V1 from each injection was quantified (volume of fluorescent pixels in V1 normalized by injection volume). See methods for further details. **B)** Our retrograde data, recalculated with FP labels. **C)** Gamanut et al. retrograde data and labels. FP labels: Au: Auditory cortex; M1,2: Primary, Secondary Motor cortex; S1,2: Primary, Secondary Somatosensory cortex; A29, A30, A24: Cingulate cortex area 29,30,24; PRh: Perirhinal cortex; MEnt: Medial enthorhinal cortex; LEnt; Lateral enthorhinal cortex; Cl: Claustrum; Pas,Post,Prs: Para-, Post-, Pre-subiculum ; V2ML: Secondary visual cortex Mediolateral area; V2MM: Secondary visual cortex Mediomedial area; PtA: Parietal association cortex; V2La: Secondary visual cortex Lateralanterior area; V2Li: Secondary visual cortex lateralinferior area; V2Lp: Secondary visual cortex Lateroposterior area.

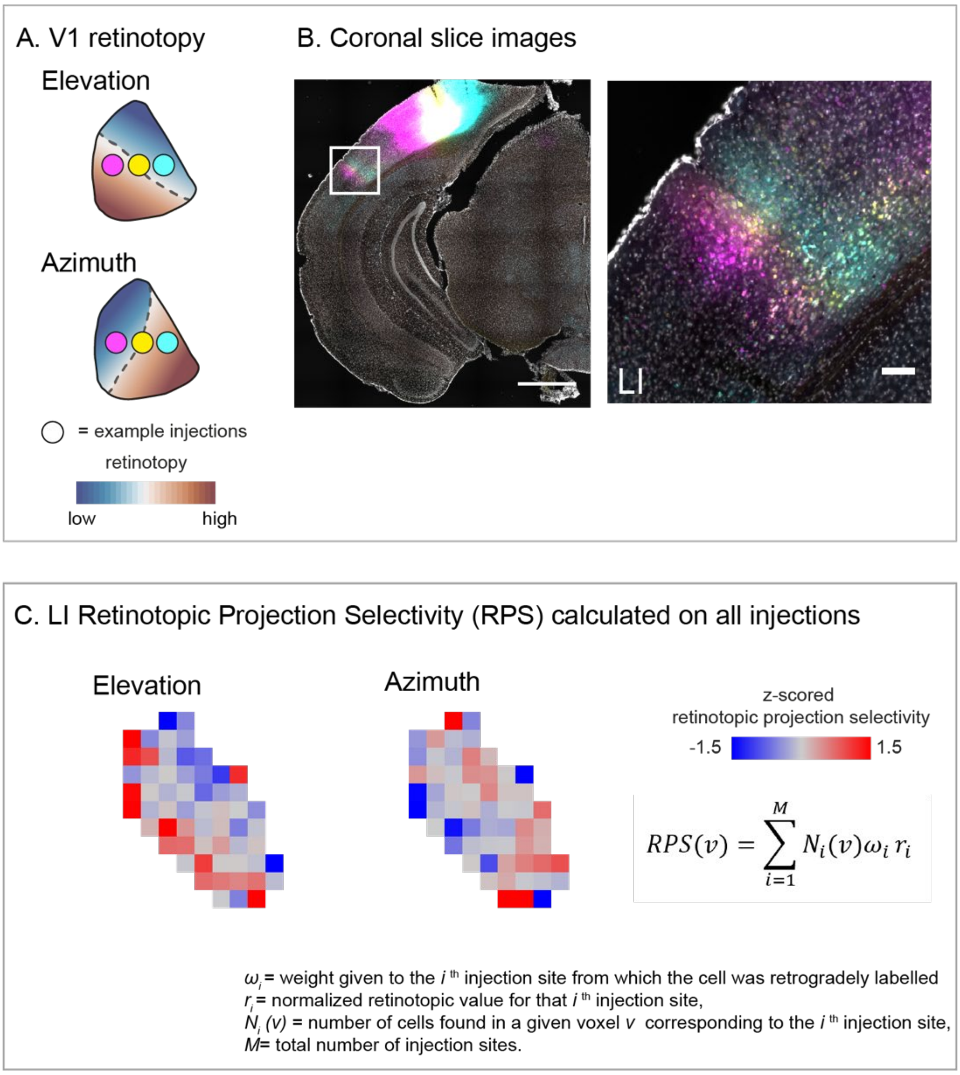

Figure S5 (related to Figure 5): Calculating retinotopic projection selectivity. A. The retinotopic map of V1 in elevation (top) and azimuth (bottom) with a schematic of the injection sites for the images in B, shown as coloured dots. **B.** Example coronal slice of animal with three injections showing retrogradely labelled cell. Right is a zoom into the area LI (same as Supplementary Figure 3C). Scale bars: left panel = 1 mm, right panel = 100 µm. **C**. Coronal projection of the retinotopic projection selectivity (RPS) for area LI calculated based on all the injections in our study. The pattern of mean RPS along the coronal slice shown in C is consistent with the multi-injection single slice image example shown in B. The expected retinotopic order of magenta, yellow and cyan cells are high to low in elevation, and low to high in azimuth (see A). This is captured by the ordering of mean RPS values in the coronal section (left to right edge).

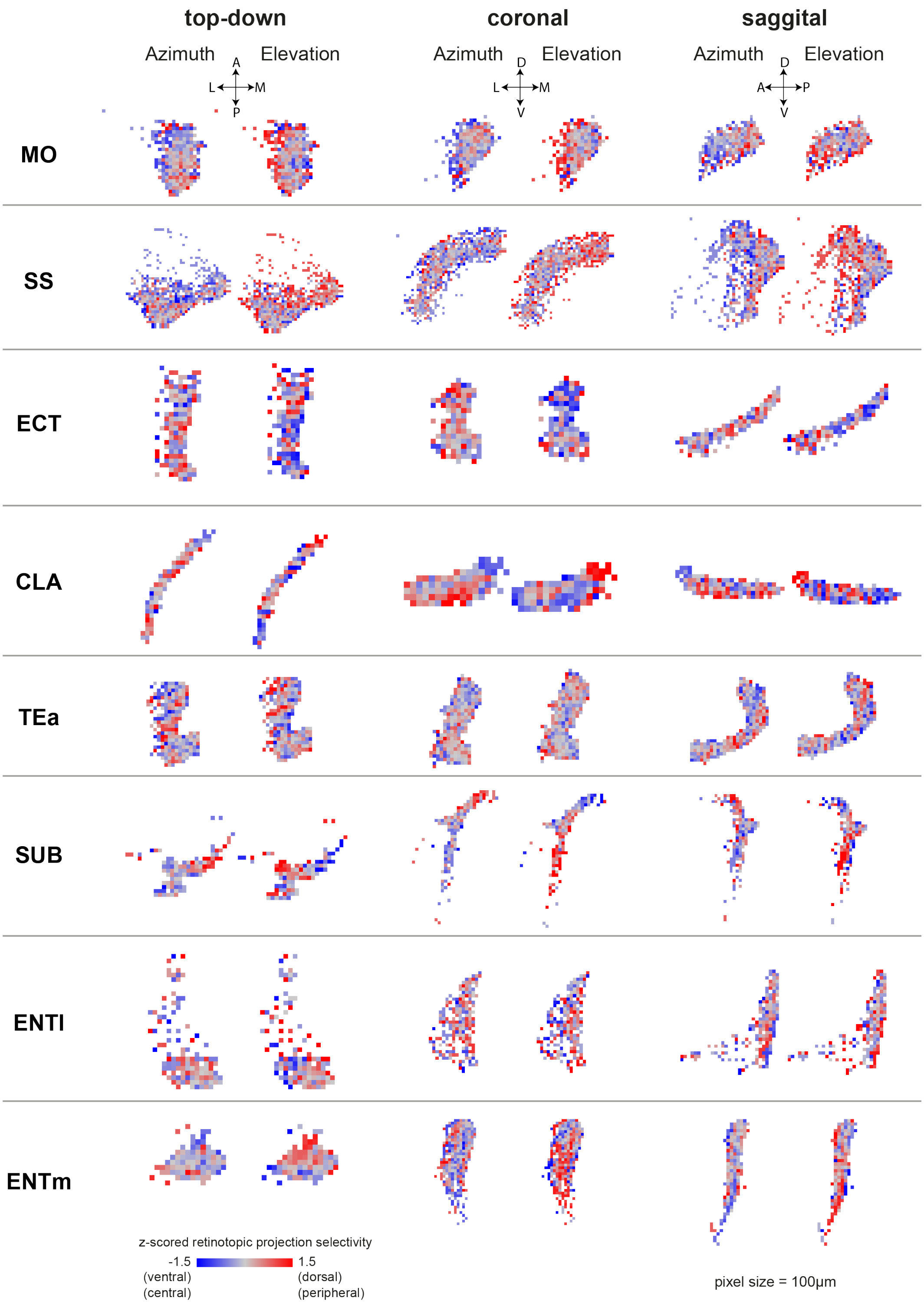

Figure S6 (related to Figure 5): Organization of cell bodies in non-visual areas projecting to V1. Each image represents one of the three views (top-down, coronal or saggital) of the 3D volume of each area. Normalized (z-scored) retinotopic projection selectivity, averaged across the top-down (left), coronal (middle) and sagittal (right) axes (binned in 100µm voxels and shown in the left hemisphere). Some non-visual areas are shown in Fig 5. Voxels are colour-coded based on their mean normalized selectivity along azimuth (left) or elevation (right) retinotopy. Only voxels that contained labelled cells are shown, causing some areas to have a sparse representation (for example SUB and ENTl).

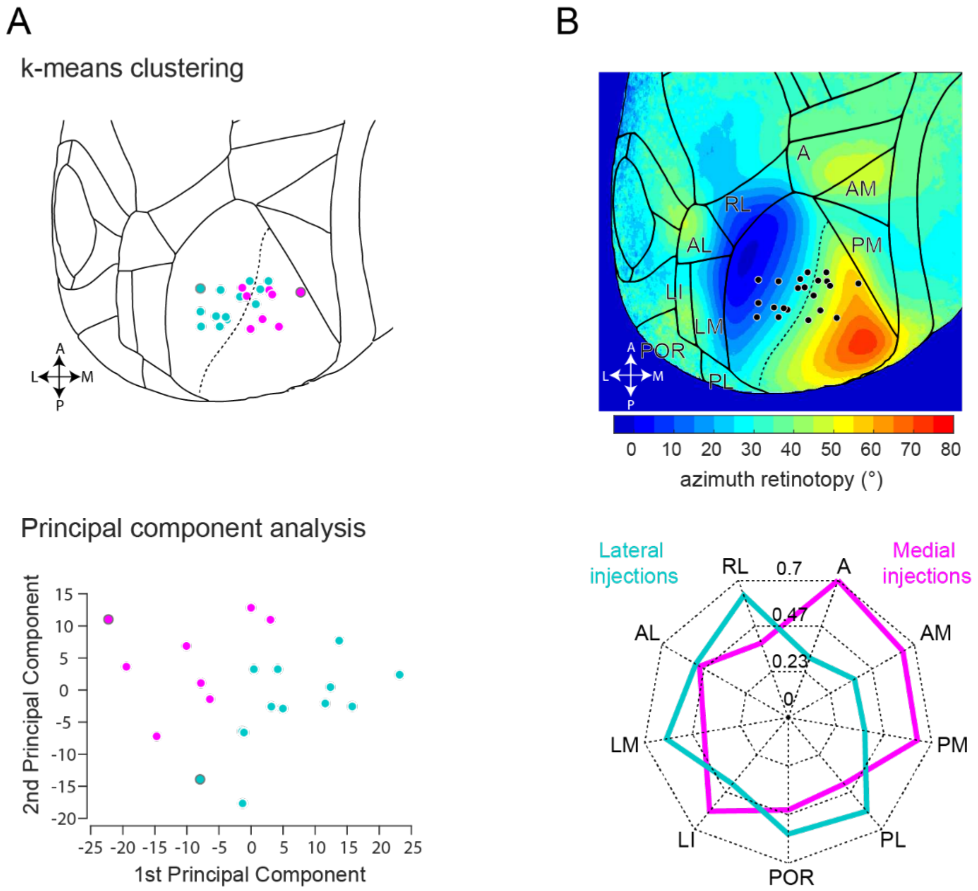

Figure S7 (related to Figure 7): A) Injection site clustering analysis: Top) k-means clustering resulted in two groups corresponding to medial and lateral portions of V1. Points with gray outline denote data points used as seeds (21 injections; 9 animals). Bottom) Applying this grouping in principal component space showed separation into two continuous groups. **B)** Injection site grouping analysis: Top) Injection coordinates plotted on mean azimuthal retinotopic map from Allen Institute. Contours are spaced 5° apart from −5° to 80° in azimuth. Bottom) Grouping injections into medial and lateral groups show higher cell counts in lateral HVAs for lateral injections, and in medial HVAs for medial injections.
