# Supplementary material for: Organization of feedback projections to mouse primary visual cortex
Source: iScience. 2021 Apr 17;24(5):102450. doi: 10.1016/j.isci.2021.102450 (PMC8169797; doi:10.1016/j.isci.2021.102450)
Supplement: Document S2. Article plus supplemental information [file mmc4.pdf]

iScience

CellPress
OPEN ACCESS

Article

Organization of feedback projections to mouse primary visual cortex

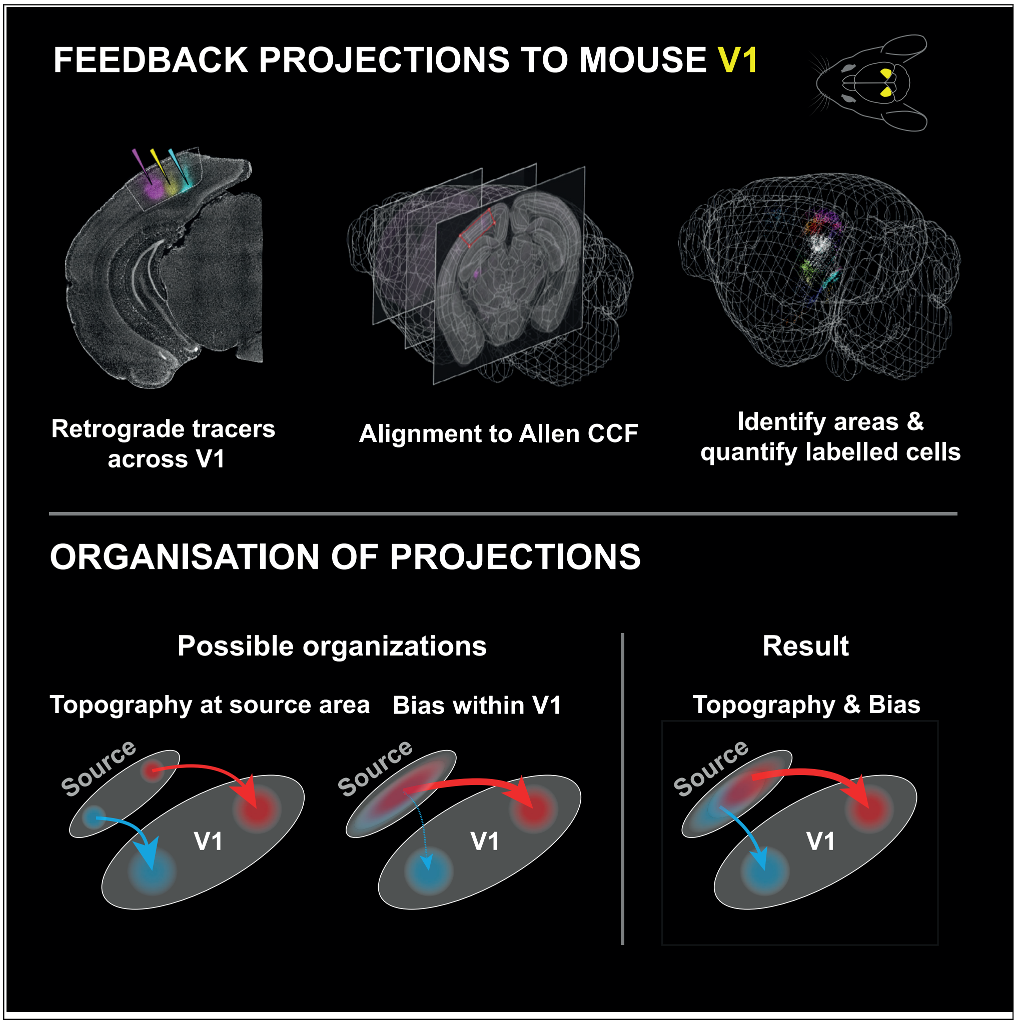

Mai M. Morimoto, Emi Uchishiba, Aman B. Saleem

m.morimoto@ucl.ac.uk (M.M.M.)
aman.saleem@ucl.ac.uk (A.B.S.)
euchishiba@gmail.com (E.U.)

Highlights

We built a software pipeline for aligning cell bodies to the standardized brain atlas

Using the pipeline, we quantified brain wide inputs to V1 using retrograde tracers

Projections from higher visual and many nonvisual areas are topographic and biased

Differential feedback inputs may be relevant for visual processing during navigation

Morimoto et al., iScience *24*, 102450
May 21, 2021 © 2021 The Authors.
https://doi.org/10.1016/j.isci.2021.102450

iScience

CellPress
OPEN ACCESS

Article

Organization of feedback projections to mouse primary visual cortex

Mai M. Morimoto,[1,2,*] Emi Uchishiba,[1,2,*] and Aman B. Saleem[1,3,*]

SUMMARY

Top-down, context-dependent modulation of visual processing has been a topic of wide interest, including in mouse primary visual cortex (V1). However, the organization of feedback projections to V1 is relatively unknown. Here, we investigated inputs to mouse V1 by injecting retrograde tracers. We developed a software pipeline that maps labeled cell bodies to corresponding brain areas in the Allen Reference Atlas. We identified more than 24 brain areas that provide inputs to V1 and quantified the relative strength of their projections. We also assessed the organization of the projections, based on either the organization of cell bodies in the source area (topography) or the distribution of projections across V1 (bias). Projections from most higher visual and some nonvisual areas to V1 showed both topography and bias. Such organization of feedback projections to V1 suggests that parts of the visual field are differentially modulated by context, which can be ethologically relevant for a navigating animal.

INTRODUCTION

Neural activity in the mouse primary visual cortex (V1) is known to be modulated by a variety of contextual signals, including arousal, locomotion, spatial context, spatial attention, or navigation (Niell and Stryker, 2010; Keller et al., 2012; Saleem et al., 2013, 2018; Vinck et al., 2015; McGinley et al., 2015; Poort et al., 2015; Fiser et al., 2016; Jurjut et al., 2017; Pakan et al., 2018; Speed et al., 2020). Perturbations of specific areas have been found to alter contextual modulations in V1. For example, optogenetic stimulation of mesencephalic locomotor region mimics the effects of locomotion in V1 (Lee et al., 2014), and optogenetic stimulation of anterior cingulate cortical projections to V1 alters sensorimotor signals in V1 (Leinweber et al., 2017). Such studies have been limited to investigating the involvement of specific areas projecting to V1. What are the other potential sources of the various contextual signals in V1? The first step toward addressing this question is knowing which areas of the brain project to V1. Here, we use an unbiased approach to quantitatively characterize brain-wide projections to V1 using retrograde tracing.

Higher visual areas (HVAs) are known to provide feedback inputs to V1 and modulate receptive field properties, especially in the surround field in mouse and other species (Nurminen et al., 2018; Vangeneugden et al., 2019; Keller et al., 2020). In mouse, around nine discrete cortical areas have been defined as HVAs based on architectonic signatures and functional properties (Wang and Burkhalter, 2007; Garrett et al., 2014; Glickfeld and Olsen, 2017; Zhuang et al., 2017). The HVAs have been categorized into two distinct streams based on their anatomical connectivity and have different distributions of spatial and temporal response properties, proposed to be analogous to the ventral and dorsal streams of primates (Andermann et al., 2011; Marshel et al., 2011; Wang et al., 2011, 2012; Tohmi et al., 2014; Glickfeld and Olsen, 2017; Murakami et al., 2017). Here, we investigated the organization of inputs from all HVAs to V1.

Continuous organizational patterns have been observed for receptive field position and various tuning properties in V1 and HVAs. Retinotopy across different HVAs has been shown to be biased: with HVAs medial to V1 generally biased to representing the peripheral visual field, whereas HVAs lateral to V1 biased to the central visual field (Garrett et al., 2014; Zhuang et al., 2017). Growing evidence points to additional functional properties being distributed topographically: including binocular disparity tuning (La Chioma et al., 2019), color tuning (Aihara et al., 2017), coherent motion processing (Sit and Goard, 2020), and orientation tuning (Fahey et al., 2019) across V1 and HVAs. How interconnections between V1 and HVAs contribute to these mesoscale topographies is not well understood. Patterns of connectivity between V1 and HVAs shed light on possible underlying mechanisms. Projection-specific calcium

[1]UCL Institute of Behavioural Neuroscience, Department of Experimental Psychology, University College London, London, WC1H 0AP, UK

[2]These authors contributed equally

[3]Lead contact

*Correspondence: m.morimoto@ucl.ac.uk (M.M.M.), aman.saleem@ucl.ac.uk (A.B.S.), euchishiba@gmail.com (E.U.)

https://doi.org/10.1016/j.isci.2021.102450

© 2021 The Authors.
This is an open access article under the CC BY license (http://creativecommons.org/licenses/by/4.0/).

imaging studies have revealed reciprocal connections at a single-cell level: feedforward projection cells from V1 match tuning properties of recipient HVAs (Glickfeld et al., 2013; Huh et al., 2018), and in turn, V1 cells receive a large portion of their feedback inputs from the HVA they project to (Kim et al., 2020). Anatomical tracing of feedforward projections from V1 to HVAs are known to show striking topography (Wang and Burkhalter, 2007), and connectivity between V1 and thalamic regions, dorsal lateral geniculate nucleus (LGN) (Song et al., 2020) and lateral posterior nucleus (LP) (Bennett et al., 2019; Juavinett et al., 2020), are known to be topographic. Compared with feedforward projections, feedback projections to mouse V1 have not been studied as comprehensively. Some studies using anterograde tracers and projection-specific functional imaging have found topographic projections to V1 from a few HVAs (lateromedial [LM], anterolateral [AL]) (Wang et al., 2011; Marques et al., 2018; Keller et al., 2020) and anterior cingulate cortex (ACA) (Leinweber et al., 2017). However, the organization of feedback inputs to V1 from across the brain has not been fully characterized.

In this study, we investigated inputs to V1 using the retrograde tracer cholera toxin subunit B (CTB). To quantify the inputs to V1 from different brain areas, the first step was to detect labeled cells and identify the brain areas where they are present. As most existing software tools (Fürth et al., 2018; Song et al., 2020; Tyson et al., 2021) require extensive setup and training, we developed a simple and modular software pipeline to quantify labeled cells across the brain. Our pipeline takes 2-dimensional (2D) images collected with a standard microscope, detects cells, and aligns the images to the Allen Common Coordinate Framework (CCF), thereby allowing 3-dimensional (3D) reconstruction of cell positions. Using this software pipeline, we found inputs to V1 originating from visual thalamic nuclei, all HVAs, and more than fourteen non-visual brain areas. The number of cells that project to V1 varied across different areas and was most prominent from the retrosplenial (RSP) cortex and HVAs. Based on the distribution of cell bodies and projections in V1, we evaluated two organizational metrics, ''topography'' and ''bias.'' Inputs from many areas tended to be topographically organized, which qualitatively matched retinotopy in HVAs. Biased inputs were observed in most HVAs and some other cortical areas.

RESULTS

To investigate inputs to V1, we injected a retrograde tracer, CTB conjugated with Alexa Fluor (488, 555 or 647), in 2 or 3 sites across the extent of V1 in each mouse (Figures 1A and S1; Table S2). CTB is known to be taken up mainly by axon terminals at the injection site and primarily retrogradely labels neuronal cell bodies (Conte et al., 2009; Nassi et al., 2015). In addition to its robust uptake, transport, and low toxicity, CTB was especially suitable to use for our multicolor injections compared with some viral tracers that are known to exhibit superinfection interference (Ohara et al., 2009). Two weeks after injection, we sectioned coronal brain slices, stained them with DAPI, and obtained images across the anterior-posterior extent of the brain using standard fluorescent light microscopes (Leica DMi8 or Zeiss Axio Scan). As expected, the retrograde tracer injected into V1 labeled cell bodies of neurons across various areas of the brain, with additional labeling of some neuronal processes (Figures 1 and 2).

Software pipeline for mapping labeled cell bodies to brain areas

To identify cell bodies labeled by the tracer and quantify their occurrence across various brain areas, we developed a software pipeline that maps each labeled cell body to its corresponding brain area, based on the Allen Reference Atlas (ARA) (Wang et al., 2020). First, we found the centroid locations of the cell bodies in each brain slice by segmenting CTB-labeled cell bodies in the corresponding fluorescent channel image and extracting the centroids of these cell bodies (STAR methods, Figure S2). We used the centroid locations to generate a binary mask image (*centroid mask image*, Figure 1B). Second, we converted centroid locations to the Allen CCF. For this, we used SHARP-Track (Shamash et al., 2018) to transform our ''*DAPI image*'' (DAPI stain) to fit the Allen CCF. SHARP-Track allowed us to identify the particular slice (''*reference slice*'') within the Allen CCF 3D model brain that corresponds to our *DAPI image*. Next, we manually selected corresponding anatomical landmarks in the *DAPI image* and *reference slice* (e.g. outer edge of slice, edge of ventricles, distinct features within hippocampus, and so on; Figure 1C [i–ii]), which were used to locally transform the *DAPI image* to fit the *reference slice* (Figure 1C [iii]). The same image transformations were then applied to the *centroid mask image*, thus converting the centroid positions into Allen CCF. Finally, we identified the corresponding brain area of centroids (of each labelled cells) based on the ARA (defined in Allen CCF). Through this semiautomated procedure, we obtained area identities of the cell bodies, which could be visualized on the Allen CCF 3D model brain (Figure 1E and Video S1).

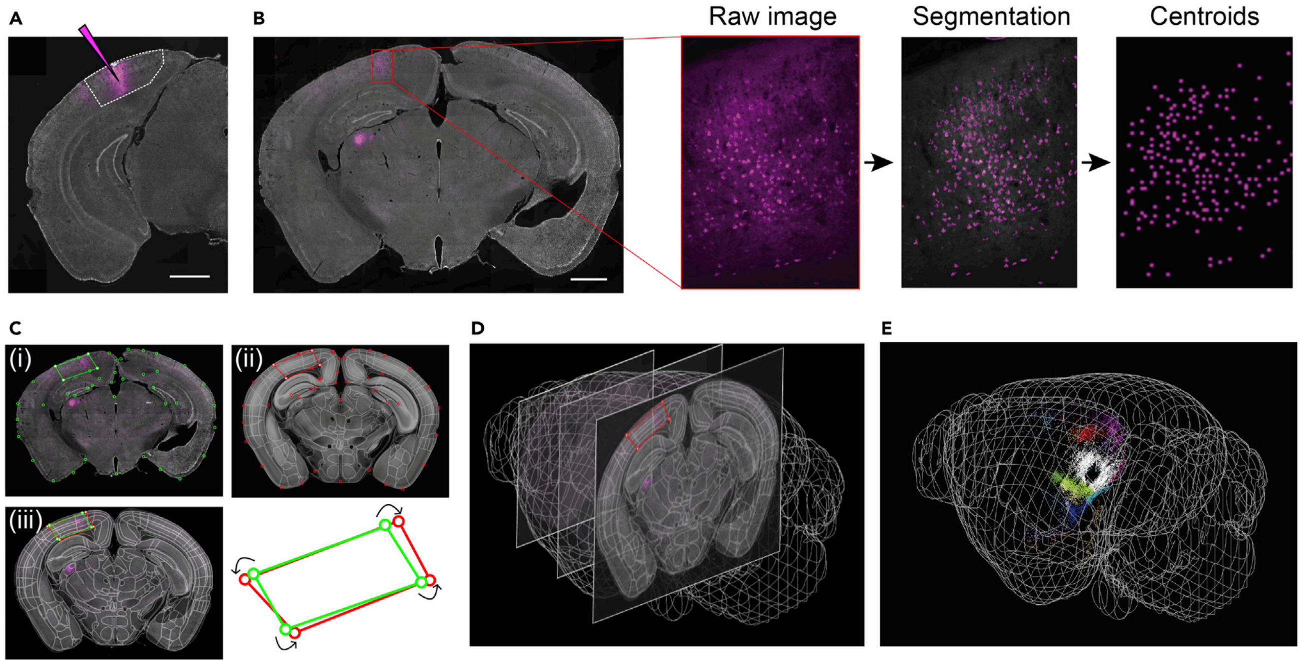

Figure 1. Software pipeline for mapping cell body locations to brain regions through alignment with Allen CCF

(A) Example injection site of CTB in V1. White dotted line denotes V1 boundary based on the Allen Reference Atlas (ARA). Scale bar: 1 mm.

(B) Demonstration of the steps used to detect labeled cells: cells were segmented from the raw image and their centroids were extracted. Scale bar: 1 mm.

(C) Images of brain slices were registered to the ARA using SHARP-Track. We illustrate an example of this procedure, where transformation points on DAPI image ([i], green) were transformed to fit a reference slice (from ARA) transformation points ([ii], red). Overlay of (i) and (ii) shown in (iii).

(D) The registration was carried out across all brain slices along the anterior-posterior axis.

(E) An example visualization of cell body locations on the 3D brain model with cells color-coded by area identity (see also Figures S1, S2 and Video S1).

HVAs, auditory cortex, and RSP cortex provide largest cortical inputs to V1

We identified more than 24 brain areas that provided inputs to V1 (Figures 2 and S3). As expected in the thalamus, we found a dense cluster of labeled cells in dorsal LGN and LP, which are visual areas known to provide inputs to V1 (Figure 2B). In the cortex, we found labeled cells in 9 HVAs, namely LM, AL, rostrolateral (RL), anterior (A), anteromedial (AM), posteromedial (PM), laterointermediate (LI), posterolateral (PL) and postrhinal (POR) (Figure 2C shows examples from PM and LI). We also found labeled cells in other cortical areas including RSP cortex, ACA, and medial entorhinal cortex (MEC) (Figure 2D). In our samples with multiple injections in the same hemisphere, the spatial arrangement of the injections was reflected in LGN, LP, and many HVAs (Figures 2B and 2C). We excluded LGN and LP from further analyses as higher-resolution imaging was required to reliably detect cells, and their strong inputs to V1 are relatively well-established. To quantify the relative number of cells that project from each brain area, we calculated the percentage of the cells in a given brain area labeled by each injection (normalizing by total number of labeled cells, excluding cells in V1, LGN, and LP). We used percentage cells per injection (all sites are shown in Figure 3A) to quantify the distribution of inputs to V1 (single example injection shown in Figure 1E) while also considering the variation in injection volume and efficacy of CTB uptake (raw cell counts across all areas are listed in Table S3).

HVAs together had the most inputs to V1, followed by the RSP, cingulate, and other sensorimotor cortices (Figure 3). The largest number of cells detected was in the HVAs (all HVAs: ~59%; median across all injections). Within the HVAs, we found the largest inputs from the LM (LM: 14.6%), followed by POR (POR: 8.6%), and PM (PM: 8.0%). In nonvisual sensorimotor areas, we found highest cell counts in auditory areas (AUD: 6.6%), followed by somatosensory (SS: 2.7%), and motor (MO: 1.6%) areas. In nonsensory areas, we found cells in the RSP cortex (16.5%), temporal association area (TEa: 5.3%), ACA (3.2%), medial entorhinal area (MEC: 1.8%), ectorhinal area (ECT: 0.6%), and lateral entorhinal area (LEC: 0.5%). In addition, we found labeled cells in nonvisual subcortical areas including the claustrum (CLA: 2.3%) and the subicular complex (SUBcom: 1.1%).

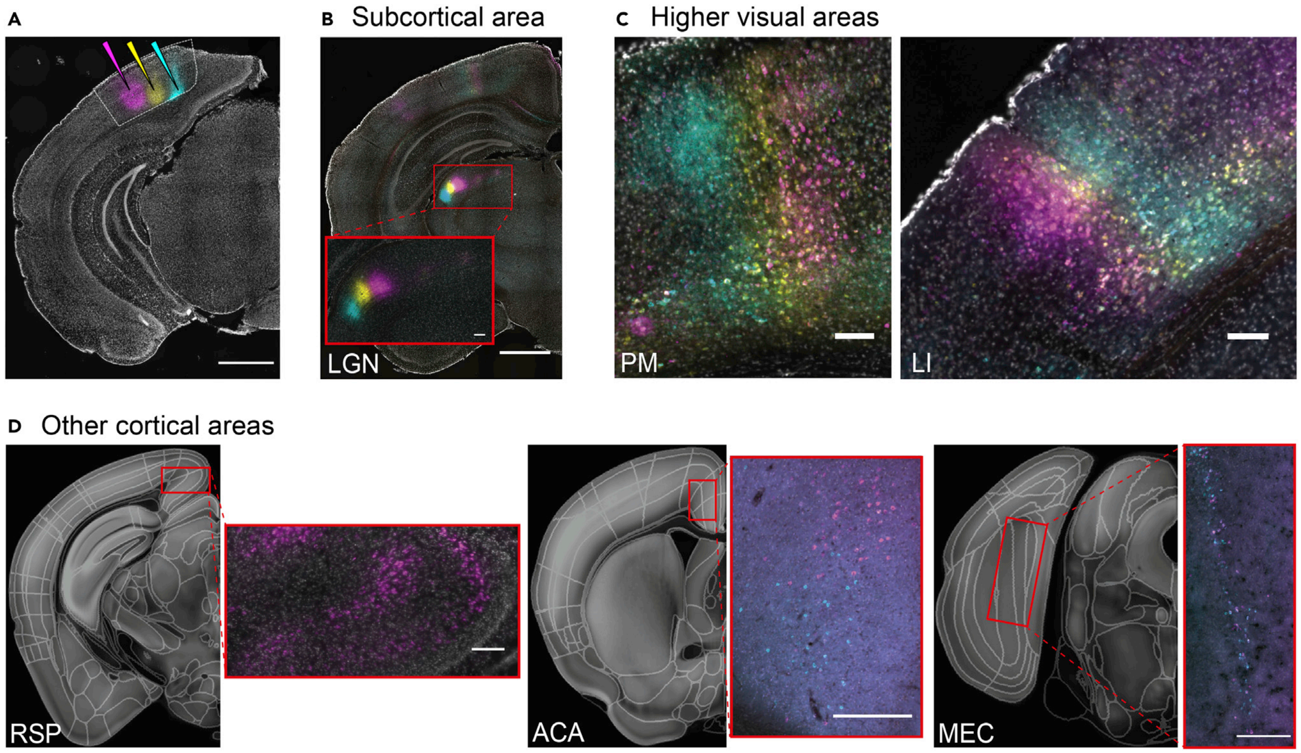

Figure 2. Retrogradely labeled cells detected in various brain regions

(A) Example of multiple CTB injections in V1 (CTB-488 [cyan], CTB-647 [yellow], and CTB-555 [magenta]).

(B–D) Example areas showing retrogradely labeled cells. Retinotopic organization of cells can be seen in LGN (B), and higher visual areas (PM and LI shown) (C). Some neural processes were also labeled in these areas. Cells were observed in other cortical areas such as RSP, ACA, and MEC (D). Right side panels correspond to red rectangle regions on the left panel. Scale bars: (A and B): 1 mm, (C and D): 100 μm. (B and C) are from the injections shown in (A), and images in (D) are from different injections . Area name abbreviations are in Table S1. (see also Figure S3, Table S1).

The distribution of cells observed with our retrograde tracing methods was broadly consistent with results from anterograde tracing and other brain atlases (Figure S4). To verify that our findings, based on retrograde tracing and CCFv3 boundaries, are not particular to these conditions, we performed three different controls. First, we used anterograde tracing data from the Allen Brain Connectivity Atlas to infer the strength of projections into V1. We found a similar distribution of projections, with HVAs being the dominant source of inputs to V1. Other areas providing inputs to V1 included nonvisual sensorimotor areas, RSP cortex, and cingulate cortex (Figure S4A). Note that quantifying the strength of innervation (axon terminals) with anterograde tracing can be noisy owing to axons of passage or thin axons below detection threshold. In addition, there was limited sampling as we only considered areas that had injections localized to within the area (see STAR Methods for criteria). As a second control, we used retrograde tracing data from a previous study that used area definitions based on parvalbumin positive cell density and immunofluorescence of several receptors on a flattened cortex (Gămănuţ et al., 2018). The distribution of projections across areas was broadly consistent within the available data (Figure S4C). The main difference was that HVAs and RSP provided a much larger proportion of the total input to V1 compared with other areas that was observed in our data. As a third control, we used the boundary definitions based on the Franklin and Paxinos (FP) atlas, taking advantage of a recent unified atlas (Chon et al., 2019) to convert the cell labels from CCFv3 to FP labels, and recalculated the distribution of cells (Figure S4B). We found the general tendency for HVAs (V2), auditory cortex (Au), retrosplenial cortex (A29, A30), and TEa having the highest cell counts to be unchanged. However, owing to the differences in border definitions, more cells were attributed to TeA than retrosplenial areas in the FP atlas, and less than half of the cells assigned to TEa, POST, PAR, ENT, VISa and VISli in CCFv3 were assigned to a corresponding area in the FP atlas (Table comparing cell counts between CCFv3 and FP atlases is available online). Overall, while these results show a qualitative agreement, they also reflect how the results can be reliant on the definition of borders between areas, which will evolve with new information. However, similar to the approach we used to convert CCFv3 labels to FP labels, the

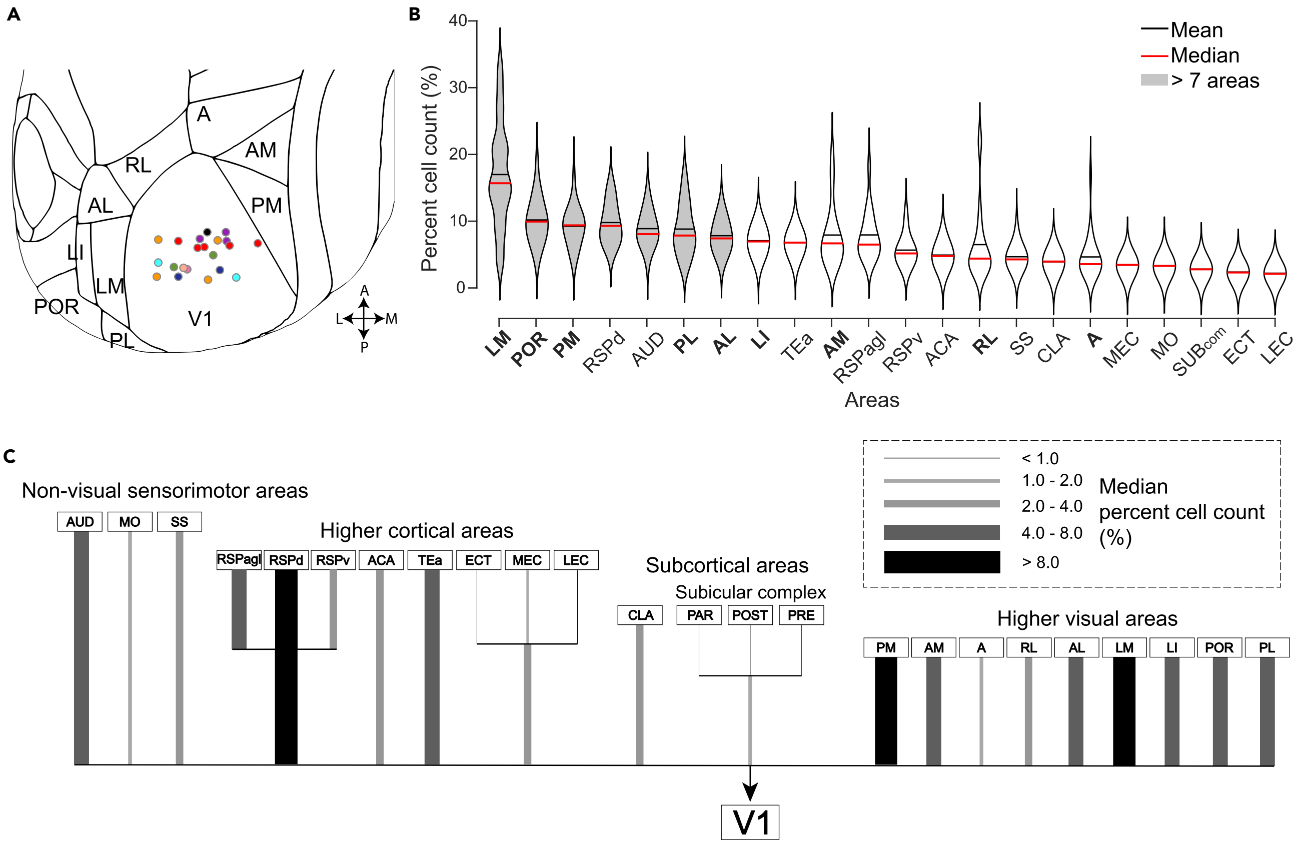

Figure 3. Distribution of inputs to V1

(A) Coordinates of all injection sites registered to the Allen Reference Atlas (21 injections; 9 animals). The color of the dots represents which animal each injection belongs to.

(B) Distribution of percentage cell counts per area pooling all injections. (16 injections; 7 animals; mean: black line, median: red line). Gray fill indicates areas with significantly higher cell count than 7 other areas (Kruskal-Wallis, multiple comparison of mean ranks, $p < 0.05$). HVA names are highlighted in bold. Area name abbreviations are listed in Table S1. Violin plots show estimated distribution of the data using normal kernels. Mean shown in black line, median shown in red line.

(C) Data in (B) illustrated as a tree diagram. Thickness and grayscale of lines relate to the median of percentage cell counts (%) as shown in legend. LGN and LP were excluded in analyses. In most areas (except RSP), results are pooled across all sub-area categories (e.g. MO = MOs + MOp, ACA = ACAd + ACAv etc.) (see also Figure S4 and Tables S1, S2, and S3).

distributions of cell bodies across regions can be recalculated based on new area definitions with the 3D coordinates that we have made available online (see STAR Methods for details).

Organization of projections to V1

As our injections into V1 spanned a range of positions in V1 (Figure 3A and Table S2), we asked if there was any organization of the inputs across V1. We define two types of organization metrics that we could evaluate with our retrograde tracing data, illustrated in Figure 4. The first measure is "*Topography*" – the organization of the cell bodies at the source area. We consider an area to have high *topography* when cell bodies labeled by different injections are localized to nonoverlapping sections of the source area, and low *topography* when they are widely distributed across overlapping sections of the source area. The second measure is "*Bias*" – the organization of the projections at the target area (i.e. V1). We consider an area to have high *bias* when it selectively projects to one part of the target area and low *bias* when it projects homogeneously across the target area.

Organization of cell bodies projecting to V1 within a source area

Cell bodies in HVAs tended to be topographically organized. We binned the source area into 100 μm voxels, and calculated a measure of topographic selectivity in each voxel we term retinotopic projection

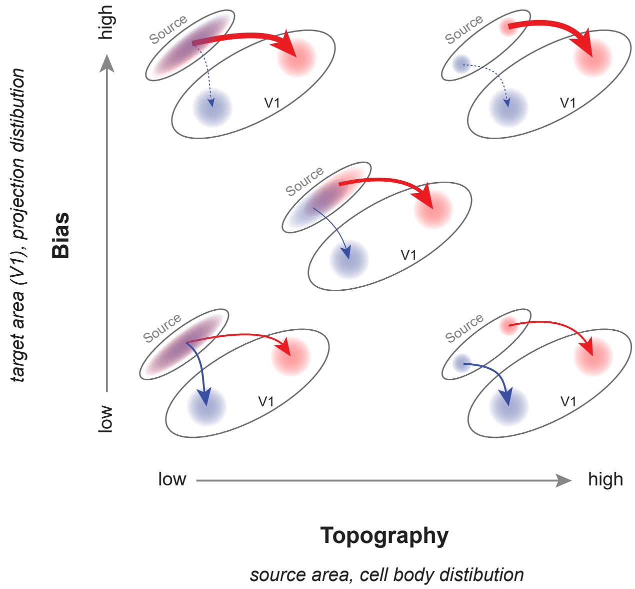

Figure 4. Possible organizations of projections to V1 that can be inferred from retrograde tracing

Description of organization of projections based on "topography" (x axis) and "bias" (y axis). We consider an area to have high topography when cell bodies labeled by different injections are localized to nonoverlapping sections of the source area, and low topography when they are widely distributed across overlapping sections of the source area. We consider an area to have high bias (indicated by the thicker red arrow and thinner blue arrow) when the fraction of cells projecting to different portion of the target area (i.e. V1) is very different and low bias when the fraction of cells projecting to different portions of the target area is similar (indicated by equal thicknesses of red and blue arrows).

selectivity (RPS). RPS is based on an estimate of the retinotopic projection target of a voxel, which we calculated by weighting the normalized retinotopic position of each injection site by the number of cells it labeled within the voxel (see STAR Methods and Figure S5 for details). We then obtained a top-down map of RPS in cortical source areas, by calculating the mean along the depth (dorsoventral axis) of the 3D volume (Figure 5). We also calculated the azimuthal and elevation maps based on a data set shared by the Allen Brain Institute (Waters et al., 2019). In both azimuthal and elevation retinotopy, top-down maps of HVAs exhibited more retinotopically selective regions (nongrey regions) that resembled the retinotopic maps of the corresponding HVA, suggesting a topographic organization of feedback projections. A similar organization was visible in some multisite injection images (Figure 2, S2, and S5). Interestingly, many nonvisual areas also showed some topography in their projections to V1 (Figures 5 and S6). However, the fraction of voxels that showed significant projection selectivity was lower in most nonvisual areas compared with HVAs (Table S4). This mild topography of projections found in nonvisual areas may suggest hitherto unknown (except ACA [Leinweber et al., 2017]) functional topography in some of these areas.

Projections to V1 from cortical areas were predominantly from deeper layers (L5, L6) compared with superficial layers (L1, L2/3) of the same area, based on CCFv3 layer definitions (Table S6). The relative percentage of supragranular (superficial layer) labeled neurons (%SLN) have been described to correlate with hierarchy of cortical areas in primates (Markov et al., 2014), where a %SLN lower than 50% is considered higher in hierarchy than the target region and thus providing feedback inputs. For most cortical areas projecting to mouse V1, we found %SLN to be lower than 25% (Table S6), which is consistent with these areas providing feedback inputs to V1. The precise value of %SLN, however, did not reflect the any of the proposed schemes of cortical hierarchy in mice (Gămănuţ et al., 2018; Harris et al., 2019; D'Souza et al., 2020), and therefore, the interpretation of %SLN for precise hierarchical order of cortical areas used in primates may need to be modified for mice.

Organization of projections within V1

We next analyzed the distribution of projections targets from a given source area, across anatomical space. For each source area, we calculated the percentage in the source area cells labeled by each injection and analyzed how this percentage varied with the anatomical locations of the injection sites (Figure 6). While the distribution of projections from some HVAs exhibited a clear bias (PM, LM, LI, POR), some areas'

CellPress
OPEN ACCESS

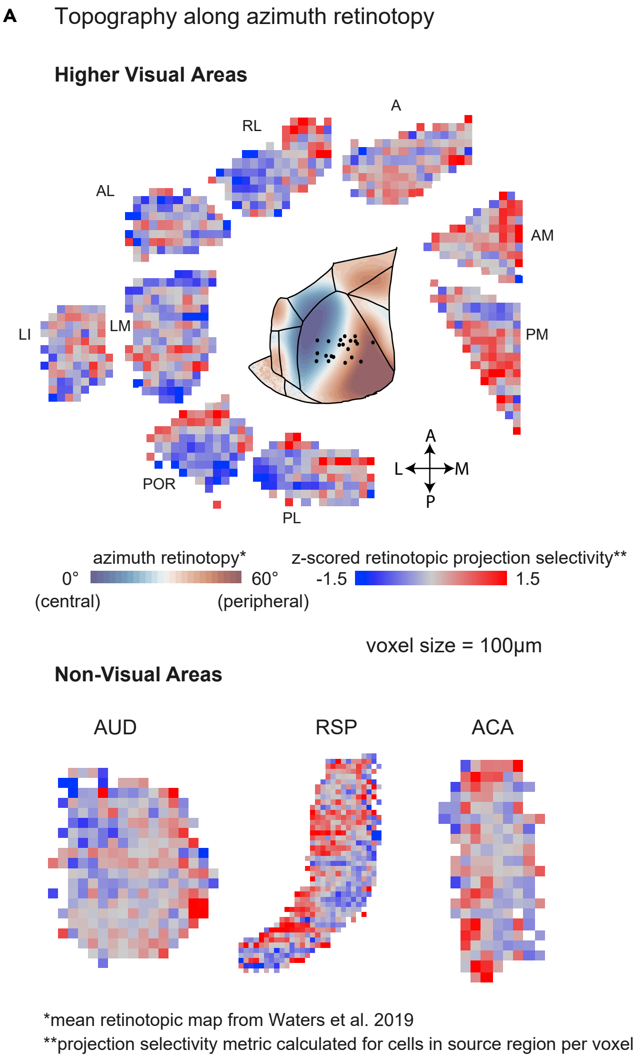

Figure 5. Organization of source area cell bodies projecting to V1

Normalized (z-scored, see Methods) "retinotopic projection selectivity" of cell bodies were binned into 100 μm voxels, averaged across the depth of cortex, shown for all higher visual areas (top), and auditory (AUD), retrosplenial (RSP) and anterior cingulate (ACA) cortices (bottom). The voxels have been color-coded based on their mean normalized selectivity along (A) azimuth or (B) elevation retinotopy. The colormap (dark blue - gray - dark red) in the center image in top panels correspond to retinotopy in azimuth (0°–60°) and elevation (−10°–15°). All areas are shown in left hemisphere orientation (see also Figure S5 and S6, Table S4).

projections appeared more homogeneous (Figure 6A). The areas that showed a bias tended to be biased toward the anatomical location of the source area, but LI was a notable exception that showed the reverse bias, away from the source area.

To quantify the organization of inputs within V1, we assessed the relationship between the retinotopic location of injection sites and distribution of cell counts in each HVA by plotting the estimated azimuthal and elevation coordinates of all injection sites against their corresponding cell counts (Figure 6B). We observed significant correlations between the cell counts and azimuth and elevation retinotopy across many HVAs, with predominantly positive correlations for HVAs that were medial to V1 (PM, AM, A) and negative correlations for most lateral areas (RL, POR, PL, with the exception of LI). Most nonvisual areas were not significantly biased, with the exceptions of RSP, POST, and ECT (Figure 6A; Table S4).

To uncover potential grouping in the projection patterns, we clustered the injection sites based on the distribution of cell counts in HVAs (and no information regarding the injection location) by considering each

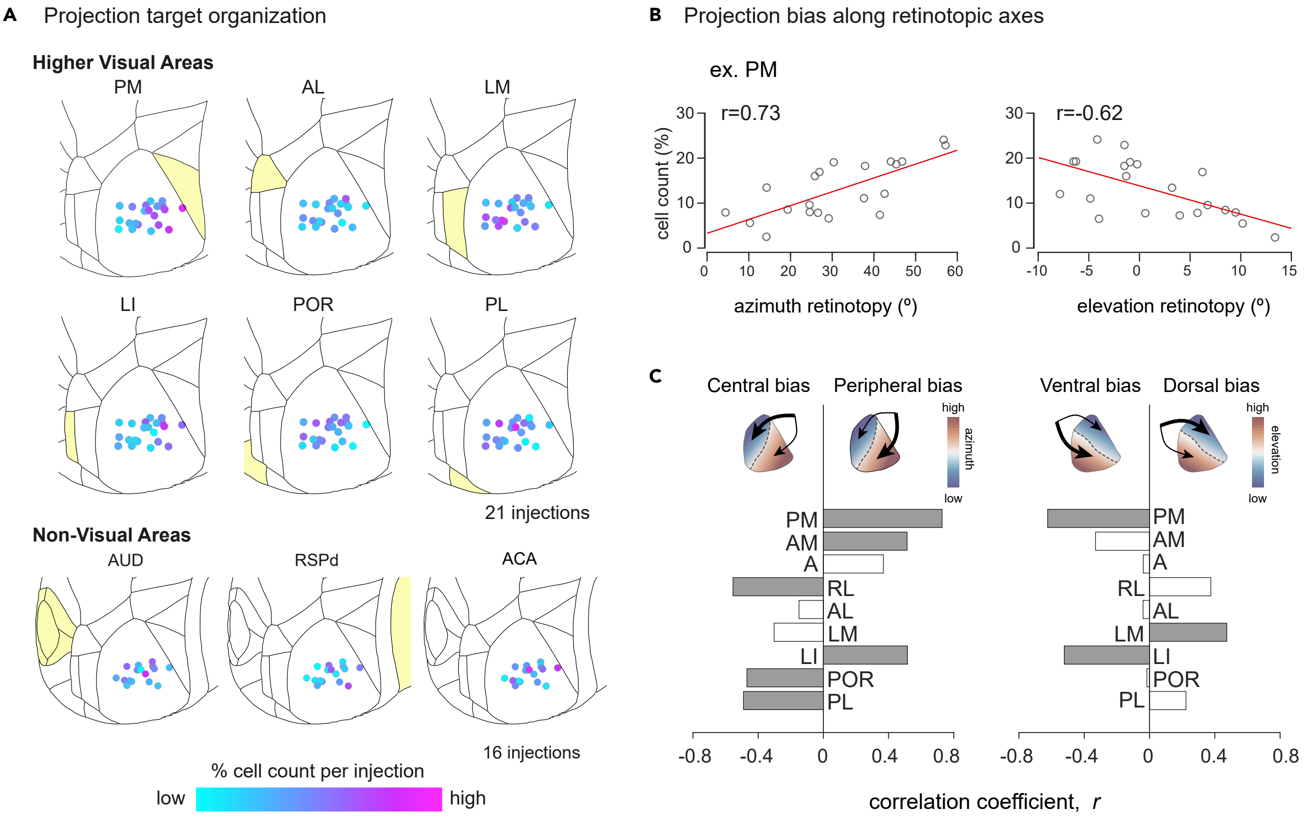

Figure 6. Organization of projections within the target area (V1)

(A) Injection sites in V1 color-coded based on normalized cell counts (% cell count) from example source areas (the range of % cell counts for each area is reported in Figure 3B). The corresponding source area is shaded in light yellow (except for ACA, which is anterior to the displayed cortical surface).

(B and C) Correlation of normalized cell counts to retinotopic axes for assessing projection bias. (B) Example correlation plot for area PM. PM showed significant correlation along both azimuth (left) and elevation (right) axes. (C) Correlation coefficients for all higher visual areas. Filled bars indicate significant correlations ($p < 0.05$). Top schematics illustrate the retinotopy (blue-red color map) and projection bias (black arrows) within V1 (see also Tables S5 and S6).

injection site as an independent data point. We used k-means clustering and classified the data into two clusters. The clustering resulted in the injection sites being grouped into roughly medial and lateral clusters in anatomical space, even though no information about the injection site was used for clustering (Figure S7A). The line of separation between the clusters can be seen when we plot the first two principal components of the distributions of HVA cells counts (Figure S7A). Splitting the data into additional clusters did not qualitatively reveal further segregation. Therefore, injection sites broadly clustered into two groups, based on the distribution of inputs from different HVAs.

The grouping of injection sites based on distribution of cell counts in HVAs was consistent with a separation along retinotopy: into sites within the central visual field and the peripheral visual field. The separation into medial and lateral clusters was roughly along the 37.5° *iso*-azimuth line, which segregated the injection sites based on whether they were in the central or peripheral visual fields. Note that the clustering into two groups along the azimuth axis could potentially reflect the larger variance of injection sites along this axis, and more focal and varied injections might reveal more refined clustering. We grouped the injections based on being below (medial) or above (lateral) 37.5°. Based on this grouping, we found that medial injections had preferentially higher cell counts in medial HVAs (A, AM, PM), and lateral injections had higher cell counts in lateral HVAs (LM, RL, PL, POR) (Figure S6B). We next analyzed inputs to V1 from all brain areas using the split of injection sites into medial and lateral groups (Figures 7A and 7B). We found minor variations in input pattern across nonvisual brain areas (Figure 7C). Only HVAs, specifically RL, AL, LM, POR and PL, showed a significant difference between medial and lateral injection groups (Figure 7C). Therefore, both correlation and clustering results indicate that there is a difference in the organization of projections along the retinotopic axes of V1, which suggests that there is a biased organization of feedback inputs from HVAs to V1.

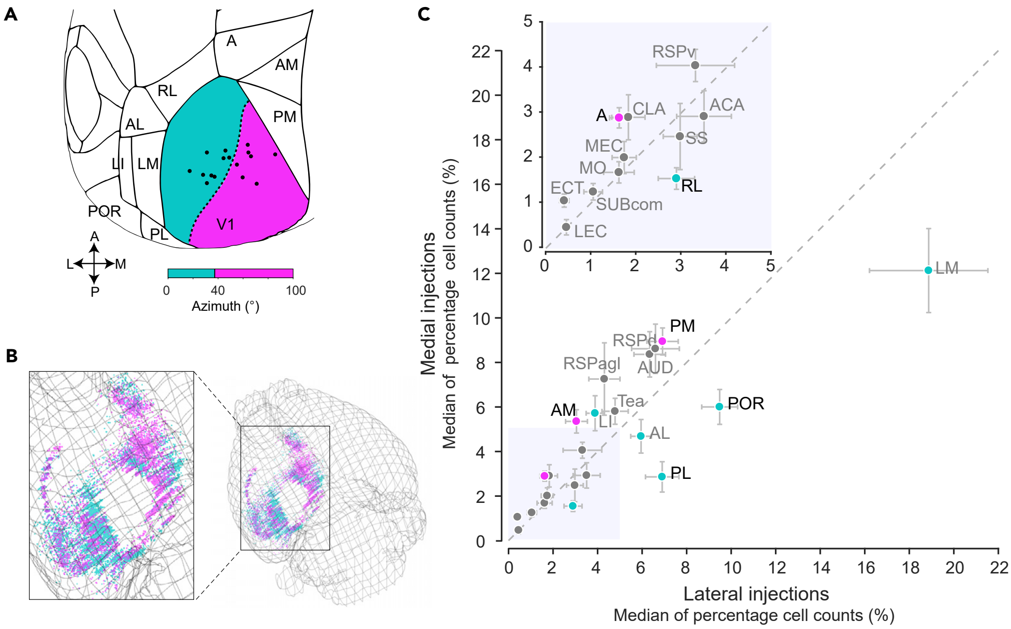

Figure 7. Differential inputs to medial versus lateral V1

(A) Grouping of injections into medial and lateral groups based on retinotopic coordinates (16 injections; 7 animals). Lateral group indicated in cyan (azimuth 0°–37.5°), medial group indicated in magenta (azimuth 37.5°–100°).

(B) Example 3D reconstruction of cells labeled with double injections (M19117) to one hemisphere (except V1, LGN, and LP). Cells labeled by the lateral V1 injection are shown in cyan, while those labeled by the medial V1 injection are shown in magenta.

(C) Median of normalized cell counts (%), medial and lateral injection groups plotted against each other. Error bars indicate median +/− S.E.M. Gray dotted line indicates the unity line. Lateral HVAs in cyan and medial HVAs in magenta. Names of areas with significant difference between medial and lateral injection groups are in black (Wilcoxon rank sum test, $p < 0.05$). Shaded region (0–5) enlarged in inset (see also Figure S7).

DISCUSSION

In this study, we investigated cortex-wide inputs to mouse V1 using the retrograde tracer CTB. We first developed a simple and modular software pipeline for the quantification of labeled cell bodies in Allen CCF, which identifies the brain area of each labeled cell. Confirming previous reports and also in agreement with anterograde data from the Allen Brain Institute, we observed cortex-wide inputs to V1, including HVAs, RSP cortex, other sensory cortices, cingulate cortex, and rhinal areas. We next quantified inputs from across the brain. The most inputs, approximately 60% of detected cells, were from HVAs. Within HVA cells, we discovered a topographic source cell organization that matched qualitatively with retinotopy. In other areas we found varying degrees of topography, but generally lower than that of HVAs. Biased projection across V1 was observed mainly from HVAs but also from other cortical areas.

Diverse areas projecting to the primary visual cortex

We developed a software pipeline to quantify and annotate cells based on the now-standard mouse brain map, ARA (Wang et al., 2020). While other software has recently been developed for this purpose (Fürth et al., 2018; Song et al., 2020; Tyson et al., 2021), our strategy is simple and modular, with little learning curve. It is semiautomated and requires human curation for cell detection and alignment to the Allen CCF. Our software takes advantage of SHARP-Track (Shamash et al., 2018) and has been developed as an extension of that framework, so that the same software package can be used for tracking electrode positions and detecting cell bodies.

We found fourteen unique brain areas that project to V1, which are not typically considered visual. These results suggest that top-down inputs associated with V1 activity might be routed directly from these areas rather than indirectly through HVAs. For example, we find inputs from the AUD, which might be involved in generating the multisensory integration observed in V1 (Iurilli et al., 2012). Inputs from the ACA have been

attributed to modulate locomotor signals (Fiser et al., 2016; Leinweber et al., 2017), and those from the CLA could be involved in change detection or modulating the salience of visual cues (Brown et al., 2017; Atlan et al., 2018). Spatial signals modulating V1 activity (Pakan et al., 2018; Saleem et al., 2018; Fournier et al., 2020) could be routed through areas that are also known to have spatial signals, including the RSP cortex (Mao et al., 2018; Nitzan et al., 2020), or the entorhinal and subicular areas (Witter et al., 2017).

We detected high cell counts in HVAs, especially LM, PM, AL and POR, dorsal RSP cortex (RSPd), and auditory cortex (AUD). This ranking of inputs per area is consistent with previous studies using retrograde tracing in cortex, albeit minor differences (Figure S4) that may have been caused by use of different reference atlases (Zingg et al., 2014; Leinweber et al., 2017; Gămănuţ et al., 2018).

Relationship between topography, bias, and retinotopic maps

Our results suggest a correlation between the mean retinotopy in HVAs and RPS based on visual comparison. However, we did not directly compare and quantify the relationship between retinotopic organization of HVAs and the retinotopic selectivity index owing to the potential for errors. The main reason for this is because we inferred retinotopy of each injection site based on a mean retinotopic map across multiple animals from an independent data set (Waters et al., 2019). Furthermore, the retinotopic map can be variable between animals (Waters et al., 2019), and our injection site covers a range retinotopic positions. Therefore, to precisely compare the two measures, we would have to measure retinotopy within an area and the retinotopy of projections from the HVAs. Using this methodology some previous studies using projection specific imaging found such correlations in LM and AL (Marques et al., 2018; Keller et al., 2020). Our data qualitatively suggest that similar correlations are likely to be present across different HVAs.

If feedback projection from HVAs to V1 followed a strict like-to-like rule (where HVA cells project only to the V1 region with matched retinotopy), we can expect to observe topography in feedback projections. Combined with the biased coverage of the visual field of HVAs, like-to-like connectivity can also lead to bias in feedback projection. Our results show that both topography and bias are present to some extent across HVAs and seem to be related to retinotopy. However, given the limitations of our method (see later section "Limitation of the study"), it is not possible to conclude whether feedback projections from HVAs to V1 follows a strict like-to-like rule from our study. Other work using functional imaging of specific feedback projections (Marques et al., 2018) observed that feedback projections from LM to V1 have a broader RF compared to that of V1, suggesting that retinotopic like-to-like connectivity might be a general but not a strict rule for feedback projections. Moreover, while retinotopic like-to-like connection might explain bias in HVAs to some extent, there may be additional mechanisms underlying such bias, especially in nonvisual areas, which do not have a clear retinotopic representation.

Organization of feedback inputs from HVAs

We observed that inputs from HVAs to V1 are topographically organized such that the distribution of inputs varies along the surface of V1. This is consistent with studies that investigated specific projections from HVAs to V1. For example, feedback projections from LM have matched receptive field centers to V1 cells in the vicinity of its projections (Marques et al., 2018).

In mice, ferrets and primates, it is known that feedback from HVAs show retinotopic convergence in which HVA feedback neurons represent a larger retinotopic area compared with the V1 region it provides feedback to (Angelucci et al., 2002; Cantone et al., 2005). These feedback projections are therefore likely to contribute to surround suppression in V1, which was shown to be the case using optogenetics in marmosets (Nurminen et al., 2018) and mice (Vangeneugden et al., 2019). It would be of interest for future studies to trace single-cell inputs retrogradely to understand the precise topography of HVA inputs to V1 (Rancz et al., 2011).

A feature defining HVAs is that each area has a retinotopic representation of the visual space or retinotopic map. The retinotopic maps in mouse HVAs have been shown to represent visual space in a biased manner, with HVAs closer together having similar biases in their representations of visual space (Zhuang et al., 2017). Overall, the biases we observed in HVAs were consistent with a general principle of medial areas preferentially projecting to medial V1 and lateral areas to lateral V1, with the exception of the area LI. This is particularly interesting given recent evidence of some functional properties of LI, specifically the preference for low-temporal and high-spatial frequencies, being similar to medial area PM (Han et al., 2018).

CellPress
OPEN ACCESS

Functional implications of biased organization of feedback inputs

Growing evidence points to functional diversity within the mouse V1 population. Binocular disparity tuning, color tuning, and coherent motion processing have recently been shown to differ along the retinotopic elevation axis of V1 (Aihara et al., 2017; La Chioma et al., 2019; Sit and Goard, 2020). In a mouse performing a task in virtual reality, task related variables were also observed to be represented continuously across visual areas (Minderer et al., 2019). These studies argue for a topographic organization of mouse visual areas at the functional level.

A diversity of functional properties across the visual field has also been observed in the primate visual system. Visual acuity and contrast sensitivity reduce with increased eccentricity in human subjects (Virsu and Rovamo, 1979; Banks et al., 1991; Duncan and Boynton, 2003), and neurons with receptive fields in the periphery have preferences for higher temporal frequencies and lower spatial frequencies in nonhuman primates (Schiller et al., 1976; De Valois et al., 1982; Foster et al., 1985; Tootell et al., 1988; Yu et al., 2010). While some of the functional differences can be explained by differential feedforward retinal input such as cortical magnification factor (Banks et al., 1991; Yu et al., 2010), some functional properties such as spatial integration (Nurminen et al., 2018) are perhaps controlled by differential feedback projections in primates (Federer et al., 2021).

The biased organization of feedback projections we observed between medial and lateral V1 might mean that V1 cells representing peripheral versus central visual fields are differentially modulated by HVAs. Ethologically, animals encounter different types of visual information in different portions of the visual field. In the case of a navigating mouse, its peripheral vision might be more frequently used for detecting optic flow, whereas the central vision might be used for landmarks to orient to. This has led to the proposal for a central and peripheral stream of processing for mouse vision (Saleem, 2020). The current finding for biased organization of HVA feedback projections to V1 is consistent with this notion. This view is further supported by recent findings that different HVA projections convey different information to V1 cells (Huh et al., 2018) and that these feedback projections follow a "like-to-like" rule in their reciprocal connection to V1 cells (Malach, 1989; Marques et al., 2018; Kim et al., 2020; Siu et al., 2020).

Conclusion

In this study, we quantified the distribution of projections to mouse V1 using a novel software pipeline. This establishes candidate brain areas and their subregions from which mouse V1 receives topographic feedback inputs, which can be targeted for observing or manipulating neural activity during various contexts. Furthermore, we found that the distribution of feedback projections from HVAs are different between the central and peripheral visual fields, consistent with the hypothesis that different portions of the visual field have different ethological demands for visual processing.

Limitations of the study

The definition of brain areas in our study was based on the ARA, coregistered to our brain images in Allen CCFv3. There are two potential errors in our methodology. The first is related to area definitions, as the precise definition of brain regions can be dependent on the atlas used (Figure S4). We can assess the atlas-dependent errors by recalculating of the distribution based on updated future atlases using coordinates of all detected cells (available online). The other error is related to the alignment of our slices to the Allen CCF. The accuracy of the alignment method was previously shown to be more reliable than localizing ROIs in an atlas by the eye (Shamash et al., 2018). While SHARP-Track can account for a certain degree of variation in the sample preparation (e.g. distortions from sectioning), there is a manual component to its alignment where anatomical landmarks are used to align slices. As these are subjective assessments based on the experimenter identifying anatomical landmarks in the DAPI images, they can lead to additional errors. However, we did verify whether known architectonic signatures in the DAPI images matched our area annotations, such as rapid thinning of layer 4 as the transition between RSP cortex and HVAs, and found them to be consistent with each other.

STAR★METHODS

Detailed methods are provided in the online version of this paper and include the following:

- KEY RESOURCES TABLE
- RESOURCE AVAILABILITY

- Lead contact
- Materials availability
- Data and code availability
- EXPERIMENTAL MODEL AND SUBJECT DETAILS
- METHOD DETAILS
  - Surgery and injection
  - Histology and imaging
- QUANTIFICATION AND STATISTICAL ANALYSIS
  - Detection and quantification of labeled cells
  - Retinotopic location of injection sites
  - Analysis of anterograde tracing data
  - Other analyses

SUPPLEMENTAL INFORMATION

Supplemental information can be found online at https://doi.org/10.1016/j.isci.2021.102450.

ACKNOWLEDGMENTS

We thank Thomas Wheatcroft for sharing code for pre-processing brain images. We also thank Stefano Zucca, Philip Shamash, Andrew MacAskill, Rawan AlSubaie, Candela Sánchez-Bellot, and Samuel Solomon for advice and useful discussions. We appreciate Jack Water's guidance on alignment of Allen Institute's retinotopic maps to our dataset. Finally, we thank all members of the lab for comments and discussions. This work was supported by the Wellcome Trust (200501), Biotechnology and Biological Sciences Research Council (R004765), HFSP (RGY0076/2018), and Royal Society (RGS\R1\191449) grants.

AUTHOR CONTRIBUTIONS

This study was conceptualized by MMM and ABS; the methodology was developed by MMM; the data was collected, curated and analyzed by MMM and EU; the visualization and software was by MMM, EU and ABS; the project was supervised by MMM and ABS; funding was acquired by ABS; and the article was written by all authors.

DECLARATION OF INTERESTS

The authors declare no conflicting interests.

Received: August 3, 2020
Revised: February 1, 2021
Accepted: April 14, 2021
Published: May 21, 2021

REFERENCES

Aihara, S., Yoshida, T., Hashimoto, T., and Ohki, K. (2017). Color representation is retinotopically biased but locally intermingled in mouse V1. Front. Neural Circuits *11*, 22.

Andermann, M.L., Kerlin, A.M., Roumis, D.K., Glickfeld, L.L., and Reid, R.C. (2011). Functional specialization of mouse higher visual cortical areas. Neuron *72*, 1025–1039.

Angelucci, A., Levitt, J.B., Walton, E.J.S., Hupe, J.-M., Bullier, J., and Lund, J.S. (2002). Circuits for local and global signal integration in primary visual cortex. J. Neurosci. *22*, 8633–8646.

Atlan, G., Terem, A., Peretz-Rivlin, N., Sehrawat, K., Gonzales, B.J., Pozner, G., Tasaka, G.-I., Goll, Y., Refaeli, R., Zviran, O., et al. (2018). The claustrum supports resilience to distraction. Curr. Biol. *28*, 2752–2762.e7.

Banks, M.S., Sekuler, A.B., and Anderson, S.J. (1991). Peripheral spatial vision: limits imposed by optics, photoreceptors, and receptor pooling. J. Opt. Soc. Am. A *8*, 1775–1787.

Bennett, C., Gale, S.D., Garrett, M.E., Newton, M.L., Callaway, E.M., Murphy, G.J., and Olsen, S.R. (2019). Higher-order thalamic circuits channel parallel streams of visual information in mice. Neuron *102*, 477–492.e5.

Brown, S.P., Mathur, B.N., Olsen, S.R., Luppi, P.-H., Bickford, M.E., and Citri, A. (2017). New breakthroughs in understanding the role of functional interactions between the neocortex and the claustrum. J. Neurosci. *37*, 10877–10881.

Cantone, G., Xiao, J., McFarlane, N., and Levitt, J.B. (2005). Feedback connections to ferret striate cortex: direct evidence for visuotopic convergence of feedback inputs. J. Comp. Neurol. *487*, 312–331.

Chon, U., Vanselow, D.J., Cheng, K.C., and Kim, Y. (2019). Enhanced and unified anatomical labeling for a common mouse brain atlas. Nat. Commun. *10*, 5067.

Conte, W.L., Kamishina, H., and Reep, R.L. (2009). Multiple neuroanatomical tract-tracing using fluorescent Alexa Fluor conjugates of cholera toxin subunit B in rats. Nat. Protoc. *4*, 1157–1166.

De Valois, R.L., Albrecht, D.G., and Thorell, L.G. (1982). Spatial frequency selectivity of cells in macaque visual cortex. Vis. Res. *22*, 545–559.

D'Souza, R.D., Wang, Q., Ji, W., Meier, A.M., Kennedy, H., Knoblauch, K., and Burkhalter, A. (2020). Canonical and noncanonical features of the mouse visual cortical hierarchy. bioRxiv. https://doi.org/10.1101/2020.03.30.016303.

Duncan, R.O., and Boynton, G.M. (2003). Cortical magnification within human primary visual cortex

correlates with acuity thresholds. Neuron 38, 659–671.

Fahey, P.G., Muhammad, T., Smith, C., Froudarakis, E., Cobos, E., Fu, J., Walker, E.Y., Yatsenko, D., Sinz, F.H., Reimer, J., and Tolias, A.S. (2019). A global map of orientation tuning in mouse visual cortex. bioRxiv. https://doi.org/10.1101/745323.

Federer, F., Ta'afua, S., Merlin, S., Hassanpour, M.S., and Angelucci, A. (2021). Stream-specific feedback inputs to the primate primary visual cortex. Nat. Commun. 12, 228.

Fiser, A., Mahringer, D., Oyibo, H.K., Petersen, A.V., Leinweber, M., and Keller, G.B. (2016). Experience-dependent spatial expectations in mouse visual cortex. Nat. Neurosci. 19, 1658–1664.

Foster, K.H., Gaska, J.P., Nagler, M., and Pollen, D.A. (1985). Spatial and temporal frequency selectivity of neurones in visual cortical areas V1 and V2 of the macaque monkey. J. Physiol. (Lond) 365, 331–363.

Fournier, J., Saleem, A.B., Diamanti, E.M., Wells, M.J., Harris, K.D., and Carandini, M. (2020). Modulation of visual cortex by hippocampal signals. Curr. Biol. 30, 3811–3817, https://doi.org/10.1016/j.cub.2020.07.006.

Fürth, D., Vaissière, T., Tzortzi, O., Xuan, Y., Märtin, A., Lazaridis, I., Spigolon, G., Fisone, G., Tomer, R., Deisseroth, K., et al. (2018). An interactive framework for whole-brain maps at cellular resolution. Nat. Neurosci. 21, 139–149.

Gămănuţ, R., Kennedy, H., Toroczkai, Z., Ercsey-Ravasz, M., Van Essen, D.C., Knoblauch, K., and Burkhalter, A. (2018). The mouse cortical connectome, characterized by an ultra-dense cortical graph, maintains specificity by distinct connectivity profiles. Neuron 97, 698–715.e10.

Garrett, M.E., Nauhaus, I., Marshel, J.H., and Callaway, E.M. (2014). Topography and areal organization of mouse visual cortex. J. Neurosci. 34, 12587–12600.

Glickfeld, L.L., Andermann, M.L., Bonin, V., and Reid, R.C. (2013). Cortico-cortical projections in mouse visual cortex are functionally target specific. Nat. Neurosci. 16, 219–226.

Glickfeld, L.L., and Olsen, S.R. (2017). Higher-order areas of the mouse visual cortex. Annu. Rev. Vis. Sci. 3, 251–273.

Han, X., Vermaercke, B., and Bonin, V. (2018). Segregated encoding of spatiotemporal features in the mouse visual cortex. bioRxiv. https://doi.org/10.1101/441014.

Harris, J.A., Mihalas, S., Hirokawa, K.E., Whitesell, J.D., Choi, H., Bernard, A., Bohn, P., Caldejon, S., Casal, L., Cho, A., et al. (2019). Hierarchical organization of cortical and thalamic connectivity. Nature 575, 195–202.

Huh, C.Y.L., Peach, J.P., Bennett, C., Vega, R.M., and Hestrin, S. (2018). Feature-specific organization of feedback pathways in mouse visual cortex. Curr. Biol. 28, 114–120.e5.

Iurilli, G., Ghezzi, D., Olcese, U., Lassi, G., Nazzaro, C., Tonini, R., Tucci, V., Benfenati, F., and Medini, P. (2012). Sound-driven synaptic inhibition in primary visual cortex. Neuron 73, 814–828.

Juavinett, A.L., Kim, E.J., Collins, H.C., and Callaway, E.M. (2020). A systematic topographical relationship between mouse lateral posterior thalamic neurons and their visual cortical projection targets. J. Comp. Neurol. 528, 95–107.

Jurjut, O., Georgieva, P., Busse, L., and Katzner, S. (2017). Learning enhances sensory processing in mouse V1 before improving behavior. J. Neurosci. 37, 6460–6474.

Kalatsky, V.A., and Stryker, M.P. (2003). New paradigm for optical imaging: temporally encoded maps of intrinsic signal. Neuron 38, 529–545.

Keller, A.J., Roth, M.M., and Scanziani, M. (2020). Feedback generates a second receptive field in neurons of the visual cortex. Nature 582, 545–549.

Keller, G.B., Bonhoeffer, T., and Hübener, M. (2012). Sensorimotor mismatch signals in primary visual cortex of the behaving mouse. Neuron 74, 809–815.

Kim, E.J., Zhang, Z., Huang, L., Ito-Cole, T., Jacobs, M.W., Juavinett, A.L., Senturk, G., Hu, M., Ku, M., Ecker, J.R., and Callaway, E.M. (2020). Extraction of distinct neuronal cell types from within a genetically continuous population. Neuron 107, 274–282.e6.

La Chioma, A., Bonhoeffer, T., and Hübener, M. (2019). Area-specific mapping of binocular disparity across mouse visual cortex. Curr. Biol. 29, 2954–2960.e5.

Lee, A.M., Hoy, J.L., Bonci, A., Wilbrecht, L., Stryker, M.P., and Niell, C.M. (2014). Identification of a brainstem circuit regulating visual cortical state in parallel with locomotion. Neuron 83, 455–466.

Leinweber, M., Ward, D.R., Sobczak, J.M., Attinger, A., and Keller, G.B. (2017). A sensorimotor circuit in mouse cortex for visual flow predictions. Neuron 95, 1420–1432.e5.

Malach, R. (1989). Patterns of connections in rat visual cortex. J. Neurosci. 9, 3741–3752.

Mao, D., Neumann, A.R., Sun, J., Bonin, V., Mohajerani, M.H., and McNaughton, B.L. (2018). Hippocampus-dependent emergence of spatial sequence coding in retrosplenial cortex. Proc. Natl. Acad. Sci. U S A 115, 8015–8018.

Markov, N.T., Vezoli, J., Chameau, P., Falchier, A., Quilodran, R., Huissoud, C., Lamy, C., Misery, P., Giroud, P., Ullman, S., et al. (2014). Anatomy of hierarchy: feedforward and feedback pathways in macaque visual cortex. J. Comp. Neurol. 522, 225–259.

Marques, T., Nguyen, J., Fioreze, G., and Petreanu, L. (2018). The functional organization of cortical feedback inputs to primary visual cortex. Nat. Neurosci. 21, 757–764.

Marshel, J.H., Garrett, M.E., Nauhaus, I., and Callaway, E.M. (2011). Functional specialization of seven mouse visual cortical areas. Neuron 72, 1040–1054.

McGinley, M.J., Vinck, M., Reimer, J., Batista-Brito, R., Zagha, E., Cadwell, C.R., Tolias, A.S., Cardin, J.A., and McCormick, D.A. (2015). Waking state: rapid variations modulate neural and behavioral responses. Neuron 87, 1143–1161.

Minderer, M., Brown, K.D., and Harvey, C.D. (2019). The spatial structure of neural encoding in mouse posterior cortex during navigation. Neuron 102, 232–248.e11.

Murakami, T., Matsui, T., and Ohki, K. (2017). Functional segregation and development of mouse higher visual areas. J. Neurosci. 37, 9424–9437.

Nassi, J.J., Cepko, C.L., Born, R.T., and Beier, K.T. (2015). Neuroanatomy goes viral! Front. Neuroanat. 9, 80.

Niell, C.M., and Stryker, M.P. (2010). Modulation of visual responses by behavioral state in mouse visual cortex. Neuron 65, 472–479.

Nitzan, N., McKenzie, S., Beed, P., English, D.F., Oldani, S., Tukker, J.J., Buzsáki, G., and Schmitz, D. (2020). Propagation of hippocampal ripples to the neocortex by way of a subiculum-retrosplenial pathway. Nat. Commun. 11, 1947.

Nurminen, L., Merlin, S., Bijanzadeh, M., Federer, F., and Angelucci, A. (2018). Top-down feedback controls spatial summation and response amplitude in primate visual cortex. Nat. Commun. 9, 2281.

Ohara, S., Inoue, K.-I., Yamada, M., Yamawaki, T., Koganezawa, N., Tsutsui, K.-I., Witter, M.P., and Iijima, T. (2009). Dual transneuronal tracing in the rat entorhinal-hippocampal circuit by intracerebral injection of recombinant rabies virus vectors. Front. Neuroanat. 3, 1.

Pakan, J.M.P., Currie, S.P., Fischer, L., and Rochefort, N.L. (2018). The impact of visual cues, reward, and motor feedback on the representation of behaviorally relevant spatial locations in primary visual cortex. Cell Rep. 24, 2521–2528.

Poort, J., Khan, A.G., Pachitariu, M., Nemri, A., Orsolic, I., Krupic, J., Bauza, M., Sahani, M., Keller, G.B., Mrsic-Flogel, T.D., and Hofer, S.B. (2015). Learning enhances sensory and multiple non-sensory representations in primary visual cortex. Neuron 86, 1478–1490.

Rancz, E.A., Franks, K.M., Schwarz, M.K., Pichler, B., Schaefer, A.T., and Margrie, T.W. (2011). Transfection via whole-cell recording in vivo: bridging single-cell physiology, genetics and connectomics. Nat. Neurosci. 14, 527–532.

Saleem, A.B. (2020). Two stream hypothesis of visual processing for navigation in mouse. Curr. Opin. Neurobiol. 64, 70–78.

Saleem, A.B., Ayaz, A., Jeffery, K.J., Harris, K.D., and Carandini, M. (2013). Integration of visual motion and locomotion in mouse visual cortex. Nat. Neurosci. 16, 1864–1869.

Saleem, A.B., Diamanti, E.M., Fournier, J., Harris, K.D., and Carandini, M. (2018). Coherent encoding of subjective spatial position in visual cortex and hippocampus. Nature 562, 124–127.

Schiller, P.H., Finlay, B.L., and Volman, S.F. (1976). Quantitative studies of single-cell properties in monkey striate cortex. III. Spatial frequency. J. Neurophysiol. 39, 1334–1351.

Shamash, P., Carandini, M., Harris, K.D., and Steinmetz, N.A. (2018). A tool for analyzing electrode tracks from slice histology. bioRxiv. https://doi.org/10.1101/447995.

Sit, K.K., and Goard, M.J. (2020). Distributed and retinotopically asymmetric processing of coherent motion in mouse visual cortex. Nat. Commun. *11*, 3565.

Siu, C., Balsor, J., Federer, F., and Angelucci, A. (2020). A direct interareal feedback-to-feedforward circuit in primate visual cortex. bioRxiv. https://doi.org/10.1101/2020.07.07.192450.

Song, J.H., Choi, W., Song, Y.-H., Kim, J.-H., Jeong, D., Lee, S.-H., and Paik, S.-B. (2020). Precise mapping of single neurons by calibrated 3D reconstruction of brain slices reveals topographic projection in mouse visual cortex. Cell Rep *31*, 107682.

Speed, A., Del Rosario, J., Mikail, N., and Haider, B. (2020). Spatial attention enhances network, cellular and subthreshold responses in mouse visual cortex. Nat. Commun. *11*, 505.

Tohmi, M., Meguro, R., Tsukano, H., Hishida, R., and Shibuki, K. (2014). The extrageniculate visual pathway generates distinct response properties in the higher visual areas of mice. Curr. Biol. *24*, 587–597.

Tootell, R.B., Silverman, M.S., Hamilton, S.L., Switkes, E., and De Valois, R.L. (1988). Functional anatomy of macaque striate cortex. V. Spatial frequency. J. Neurosci. *8*, 1610–1624.

Tyson, A., Rousseau, C.V., Niedworok, C.J., Keshavarzi, S., Tsitoura, C., Cossell, L., Strom, M., and Margrie, T.W. (2021). A deep learning algorithm for 3D cell detection in whole mouse brain image datasets (bioRxiv). https://doi.org/10.1101/2020.10.21.348771.

Vangeneugden, J., van Beest, E.H., Cohen, M.X., Lorteije, J.A.M., Mukherjee, S., Kirchberger, L., Montijn, J.S., Thamizharasu, P., Camillo, D., Levelt, C.N., et al. (2019). Activity in lateral visual areas contributes to surround suppression in awake mouse V1. Curr. Biol. *29*, 4268–4275.e7.

Vinck, M., Batista-Brito, R., Knoblich, U., and Cardin, J.A. (2015). Arousal and locomotion make distinct contributions to cortical activity patterns and visual encoding. Neuron *86*, 740–754.

Virsu, V., and Rovamo, J. (1979). Visual resolution, contrast sensitivity, and the cortical magnification factor. Exp. Brain Res. *37*, 475–494.

Wang, Q., Ding, S.L., Li, Y., Royall, J., Feng, D., Lesnar, P., Graddis, N., Naeemi, M., Facer, B., Ho, A., et al. (2020). The allen mouse brain common coordinate framework: a 3D reference atlas. Cell *181*, 936–953.e20.

Wang, Q., and Burkhalter, A. (2007). Area map of mouse visual cortex. J. Comp. Neurol. *502*, 339–357.

Wang, Q., Gao, E., and Burkhalter, A. (2011). Gateways of ventral and dorsal streams in mouse visual cortex. J. Neurosci. *31*, 1905–1918.

Wang, Q., Sporns, O., and Burkhalter, A. (2012). Network analysis of corticocortical connections reveals ventral and dorsal processing streams in mouse visual cortex. J. Neurosci. *32*, 4386–4399.

Waters, J., Lee, E., Gaudreault, N., Griffin, F., Lecoq, J., Slaughterbeck, C., Sullivan, D., Farrell, C., Perkins, J., Reid, D., et al. (2019). Biological variation in the sizes, shapes and locations of visual cortical areas in the mouse. PLoS One *14*, e0213924.

Witter, M.P., Doan, T.P., Jacobsen, B., Nilssen, E.S., and Ohara, S. (2017). Architecture of the entorhinal cortex A review of entorhinal anatomy in rodents with some comparative notes. Front. Syst. Neurosci. *11*, 46.

Yu, H.-H., Verma, R., Yang, Y., Tibballs, H.A., Lui, L.L., Reser, D.H., and Rosa, M.G.P. (2010). Spatial and temporal frequency tuning in striate cortex: functional uniformity and specializations related to receptive field eccentricity. Eur. J. Neurosci. *31*, 1043–1062.

Zhuang, J., Ng, L., Williams, D., Valley, M., Li, Y., Garrett, M., and Waters, J. (2017). An extended retinotopic map of mouse cortex. Elife *6*, e18372.

Zingg, B., Hintiryan, H., Gou, L., Song, M.Y., Bay, M., Bienkowski, M.S., Foster, N.N., Yamashita, S., Bowman, I., Toga, A.W., and Dong, H.-W. (2014). Neural networks of the mouse neocortex. Cell *156*, 1096–1111.

CellPress
OPEN ACCESS

STAR★METHODS

KEY RESOURCES TABLE

REAGENT or RESOURCE	SOURCE	IDENTIFIER
Bacterial and virus strains		
Cholera toxin subunit B (CTB) conjugated with Alexa Fluor 488	Thermo Fisher	Cat#C34775
Cholera toxin subunit B (CTB) conjugated with Alexa Fluor 555	Thermo Fisher	Cat#C34776
Cholera toxin subunit B (CTB) conjugated with Alexa Fluor 647	Thermo Fisher	Cat#C34778
Deposited data		
Retrograde tracing data	This article	https://figshare.com/projects/Organization_of_feedback_projections_to_mouse_V1_-_data_and_supplementary_tables/98237
Mouse retinotopic maps	Waters et al., 2019, supplemental material	https://journals.plos.org/plosone/article?id=10.1371/journal.pone.0213924
Anterograde tracing data	Allen Brain Institute	https://connectivity.brain-map.org/projection
Retrograde tracing data	Gămănuţ et al., 2018, supplemental material	https://www.cell.com/neuron/fulltext/S0896-6273(17)31185-6
Experimental models: organisms/strains		
M. musculus; C57BL/6J	Charles River	RRID:IMSR_JAX:000664
Software and algorithms		
FeedbackProjections2V1	This article	https://github.com/SaleemLab/FeedbackProjections2V1
SHARP-Track	Shamash et al., 2018	https://github.com/cortex-lab/allenCCF/tree/master/SHARP-Track
MATLAB R2017-R2020	Mathworks	https://uk.mathworks.com/

RESOURCE AVAILABILITY

Lead contact

Further information and requests for resources and reagents should be directed to and will be fulfilled by the lead contact Aman B Saleem (aman.saleem@ucl.ac.uk)

Materials availability

No new material was produced during this project.

Data and code availability

Original processed data and software are available at the following:

Data: *FigShare* (https://figshare.com/projects/Organization_of_feedback_projections_to_mouse_V1_-_data_and_supplementary_tables/98237).

Code: *Github* (https://github.com/SaleemLab/FeedbackProjections2V1).

Other data and code used in this study were publicly available (also see Key resources table): mouse retinotopic maps data (Waters et al., 2019, https://doi.org/10.1371/journal.pone.0213924), anterograde tracing data (Allen Brain Institute, https://connectivity.brain-map.org/projection), retrograde tracing data (Gămănuţ et al., 2018; https://doi.org/10.1016/j.neuron.2017.12.037), SHARP-Track code (Shamash et al., 2018, https://github.com/cortex-lab/allenCCF/tree/master/SHARP-Track)

EXPERIMENTAL MODEL AND SUBJECT DETAILS

All procedures were conducted in accordance with the UK Animals Scientific Procedures Act (1986). Experiments were performed at University College London under personal and project licenses released by the Home Office following appropriate ethics review. We used 9 adult male and female wild-type mice (C57BL/6J, aged 13–30 weeks) for this study. All mice were kept on 12-h light:12-h dark cycle, with food and water available *ad libitum*. Mice were singly housed after the surgical procedures.

METHOD DETAILS

Surgery and injection

Mice were anesthetized with 2% isoflurane delivered with oxygen (0.5 L/min) and placed on a heating pad to maintain the body temperature. An incision was made to the scalp along the midline to expose the injection area, and injection coordinates for V1 (listed in Table S2) were marked using a stereotaxic software (Robot Stereotaxic, Neurostar). Small craniotomies were made at these sites, and cholera toxin subunit B (CTB) conjugated with Alexa Fluor (CTB-488, -555, -647, Thermo Fisher cat#C34775, C34776, C34778) was injected using glass micropipettes. CTB was injected in 2–3 sites in V1 bilaterally (7 mice) or unilaterally (2 mice) in V1, at depths of 250 μm or 750 μm below the pia (unilateral: M19118, M19119, bilateral double: M19114, M19115, M19121, M19122, M19123 bilateral triple: M19116, M19117, see Table S2 and Figure S1 for more details). Each injection within a hemisphere was made with unique fluorophores (CTB-488, -555, and -647). At each site, we infused 200–300 nL at a flow rate of 20–40nL/min. For some injections (see Table S2 for details), an anterograde tracer (spaghetti monster, AAV1-smFP [addgene #98928]) was coinjected, but data from this tracer were not used for this study. The micropipette was left at the injection site for 5 min after the infusion completed, before being withdrawn slowly. We covered the craniotomies with Kwik-Cast (World Precision Instruments), sutured the scalp, and allowed the animals to recover for 3 days while orally administering analgesics.

Histology and imaging

Ten to fourteen days after injection, mice were anesthetized with 5% isoflurane and injected with pentobarbital intraperitoneally. Mice were transcardially perfused with 0.9% NaCl in 0.1M phosphate buffer (PB), followed by 4% paraformaldehyde (PFA). The brain was extracted and placed in 4% PFA overnight at 4°C and subsequently cryoprotected with 30% sucrose solution. The olfactory bulb and cerebellum were cut away, and brains were frozen in O.C.T. Compound (Sakura FineTek). Coronal sections of 50-μm thickness were sliced on a cryostat (Leica, CM1850 UV), on average between AP-coordinates of bregma 0.64 $\pm$ 0.30 (S.E.M.) to -4.55 ± 0.08 (S.E.M.). Slices were mounted using Vectorshield with DAPI (Vector Labs) or ProLong Diamond Antifade Mountant with DAPI (Invitrogen) and slices 150 μm apart were imaged with a standard fluorescence microscope (Leica DMi8 or ZEISS Axio Scan.Z1) using a 10× objective and standard filter sets. The contrasts of individual channels were adjusted in figures to optimize visualization.

QUANTIFICATION AND STATISTICAL ANALYSIS

Detection and quantification of labeled cells

Our analysis pipeline consisted of a cell detection procedure followed by alignment to the Allen CCF using SHARP-Track (Shamash et al., 2018). Our cell detection algorithm steps (implemented using the image processing toolbox in MATLAB, R2017; function names in italic) were as follows: (**1**) background subtraction using morphological top-hat filter ***imtophat*** (structural element: "disk," size: 4); (**2**) binarization by ***imbinarize*** using a percentile threshold (typically 99.5 to 99.8 percentile of the fluorescence intensity distribution); (3) selecting objects larger than 25 pixels in the binary image using ***bwareaopen***; (**4**) erode and dilate edges of objects using ***imerode/imdilate*** (structural element: "disk"; size: 1); (**5**) fill holes in the objects through ***imfill*** followed by (**6**) ***watershed*** to isolate connected objects; and finally, (**7**) extract centroids of objects using ***regionprops*** (property: area >20). The automatic segmentation results were checked by visual inspection for each processed slice (Figure S2). The cell-detection process segmented the cells from neuropil that were occasionally labeled using CTB. Using the centroids from the final step, we generated a centroid mask (an image that has a value of 1 at the centroid locations and 0 elsewhere) of the same size as the original image and used this and DAPI channels as input to SHARP-Track. As SHARP-Track was originally developed to analyze electrode tracks, we adapted some functions for the purpose of quantifying cells in different brain regions. The GUI in SHARP-Track allows for the user to scroll through slices of the Allen Reference Atlas (ARA) 3D brain model and visually identify the slice that best corresponds to the imaged brain slice (DAPI image). The selected ARA slice (reference slice) can be microadjusted in the AP-, ML- and

CellPress
OPEN ACCESS

DV-axes, to account for any asymmetry introduced during histological procedures. Subsequently, transformation points were selected by clicking on corresponding anatomical landmarks between the DAPI image and reference slice. These points are used to morph the DAPI image to fit the reference slice, using local transformations (Shamash et al., 2018). We placed more transform points around our areas of interest (e.g. where we found cells) such that the fit would be more accurate around the areas of interest. We then used the same transformation on the centroid mask image to map cell body centroids to the ARA. This generated a table of all detected cells with coordinates and area identities in Allen CCF (based on the ARA). We analyzed each of the imaged slices through this pipeline and calculated the cell counts from each injection site. These cell counts were generally consistent with results from manual counting of cells performed on a subset of samples. Absolute cell counts were normalized by the total number of cells per injection (excluding cells in V1, LGN, and LP). Cell counts from individual injection sites were considered independent samples for subsequent analyses. For estimating the centroid of injection sites in Allen CCF, we first used image processing methods almost identical to cell detection described previously. For each injection, we identified the injection site in the slice with the strongest fluorescence signal (Figure S1), then after segmenting the injection site as a single object, centroids were extracted using regionprops (property: area >5000). Subsequent steps to align the centroid to ARA were identical to the method described previously. The processed data are available to download at *FigShare*, and the code for the analysis pipeline described previously, at *Github*.

Retinotopic location of injection sites

To estimate the retinotopic position of our injection sites post hoc, we followed a procedure similar to a previous study (Minderer et al., 2019) and used intrinsic imaging data published by the Allen Institute (Waters et al., 2019). This data set contained mean azimuth and elevation maps in Allen CCF from 60 mice, generated using horizontal and vertical sweeping bars (Kalatsky and Stryker, 2003). Using the injection site coordinates in Allen CCF, obtained using the software pipeline mentioned previously, we directly readout values from the mean azimuthal map and used these as estimated retinotopic positions of our injection sites. While we did not experimentally confirm the individual retinotopic location of each injection site, previous work has shown that the difference in the retinotopic maps across animals is comparable with measurement error across trials within individual animals, owing to the underlying variability in the estimation of the receptive field using wide-field intrinsic imaging (Waters et al., 2019).

Analysis of anterograde tracing data

We used anterograde tracing data from the publicly available connectivity repository of the Allen Brain Institute (https://connectivity.brain-map.org/projection). Specifically, we used the "target search" functionality in the GUI provided at the website. We selected specific brain areas (shown in Figure S4) as "source" and V1 ("VISp") as the "target" and analyzed all resulting injections from all mouse strains. The data contained measured values such as the volume of fluorescent pixels (mm^3) in the target area and injection specificity. We adopted a criterion to filter these injections: selecting for further analyses only those with injection volumes over 0.1 mm^3 and injection specificity greater than 70%. We then normalized the fluorescent volume of the target area by the injection volume to account for the variability in injection volumes. This allowed us to assess the amount of specific projections from "source" areas to "target" area V1 using anterograde tracing data.

Other analyses

To cluster injections based on the distribution of inputs connections, we used k-means clustering (Figure S6). For this clustering, we used two seeds, one in medial V1 and another in lateral V1. This was necessary because seeds randomly selected from within the same group led to variable results. For correlation analysis, we used the Pearson's correlation coefficient r.

Paired t-tests, Kruskal-Wallis, and Wilcoxon rank sum tests were used to test for statistical significance. For all comparisons, we used a significance level of $p < 0.05$.

For statistical comparison in Figure 3B, we performed a nonparametric multiple comparison procedure, comparing the mean rank of areas as groups, using the Kruskal-Wallis test. No single area had significantly larger mean rank than all other 21 areas. To highlight areas with higher mean rank, we chose a criterion of "larger than 7 other areas." The complete results of multiple comparison for Figure 3B was as follows: AUD>7, MO <11, SS<4, ACA<2, ECT<13, LEC<13, MEC<10, RSPagl>5, RSPd>8, RSPv>2&<1, Tea>4,

CLA<7, SUBcom<11, PM>8, AM>5, A<7, RL>2 & <2, AL>7, L>11, LI>5, POR>10, PL>7 (area>X: larger than X other areas, area<X: smaller than X other areas).

For visualizing source cell distribution, we first calculated ''retinotopic projection selectivity (RPS).'' Each cell was assigned a weighted retinotopic value, based on properties of the injection site from which it was retrogradely labeled. The retinotopic value was obtained by locating our injection sites within the mean retinotopic map from the Allen Brain Institute (Waters et al., 2019), then normalized to values −1 to 1 (azimuth range: 0° to 60°, elevation range: −10° to 15°). The weight was % cells labeled by that injection in the source area, used as a means to control for the injection size (to avoid larger injections with higher labeled cell counts being overrepresented). We then summed this weighted retinotopic value from all cells found in each 100 μm cubic bin (voxel) to obtain the ''RPS'' per voxel. Thus, RPS for each voxel, v, can be written as:

$$RPS(v) = \sum_{i=1}^{M} N_i(v)\omega_i r_i$$

where ω_i is the weight given to the ith injection site from which the cell was retrogradely labeled, r_i is the normalized retinotopic value for that i^{th} injection site, $N_i(v)$ is the number of cells found in a given voxel v (100 μm cubic bin) corresponding to the ith injection site, and M is the total number of injection sites.

We next normalized this RPS using a shuffled distribution to obtain the z-score for each 100 μm cubic bin. To calculate the shuffled distribution, we recalculated the selectivity value in each voxel by shuffling the injection identity 100 times to get a shuffled selectivity distribution. We then used the shuffled selectivity distribution to calculate a z-scored selectivity per voxel. The 2D maps in Figure 5 were created by taking the mean of the 3D volume of the source area along the dorsoventral axis. For Table S4 we used mean absolute z-score > 1.65 and mean absolute z-score > 1.96 as criteria for $p<0.1$ and $p<0.05$, respectively. Note that absolute values close to zero can mean either there is no (or unreliable) retinotopic selectivity in the specific voxel or the voxel is selective for an intermediate retinotopic value. Therefore, RPS is only used as an assessment for the existence of topography, rather than a precise quantitative description.

All analyses were performed in MATLAB.

iScience, Volume *24*

Supplemental information

Organization of feedback projections to mouse primary visual cortex

Mai M. Morimoto, Emi Uchishiba, and Aman B. Saleem

Supplementary Tables

Table S1 (related to Figures 2, 3): Area name abbreviations used in the study. Most follow Allen CCF (v3) nomenclature (Wang et al., 2020). Exceptions are indicated in italic, and corresponding Allen CCF (v3) nomenclature shown in brackets where existing.

V1 (VISp)	Primary visual area
AUD	Auditory areas
MO	Somatomotor areas
SS	Somatosensory areas
RSP	Retrosplenial area
RSPagl	Retrosplenial area, lateral agranular part
RSPd	Retrosplenial area, dorsal part
RSPv	Retrosplenial area, ventral part
ACA	Anterior cingulate area
TEa	Temporal association areas
ECT	Ectorhinal area
MEC (ENTm)	Entorhinal area, medial part, dorsal zone
LEC (ENTl)	Entorhinal area, lateral part
CLA	Claustrum
SUBcom	Subicular complex
PAR	Parasubiculum
POST	Postsubiculum
PRE	Presubiculum
PM (VISpm)	Posteromedial visual area
AM (VISal)	Anteromedial visual area
A (VISa)	Anterior area
RL (VISrl)	Rostrolateral visual area
AL (VISal)	Anterolateral visual area
LM (VISl)	Lateromedial visual area
LI (VISli)	Laterointermediate area
POR (VISpor)	Postrhinal area
PL (VISpl)	Posterolateral visual area

Table S3 (related to Figure 3): Number of cells detected across all brain areas.

Area	% cell count			Absolute cell count	
	Mean	SEM	Median	Total cells counted	from ‘n = ’ injections
AUD	7.465	0.679	6.615	5895	16
MO	1.606	0.224	1.645	1464	16
SS	3.063	0.402	2.652	2543	16
ACA	3.332	0.433	3.172	2777	16
ECT	0.669	0.098	0.601	537	16
LEC	0.545	0.092	0.457	476	16
MEC	1.750	0.227	1.804	1558	16
RSPagl	6.484	0.954	4.975	5144	16
RSPd	8.419	0.794	7.906	6714	16
RSPv	4.107	0.534	3.588	3255	16
TEa	5.279	0.437	5.284	4220	16
CLA	2.270	0.331	2.320	2063	16
SUBcom	1.197	0.139	1.171	990	16
PM	7.837	0.648	7.988	6991	21
AM	6.467	1.066	5.163	5317	21
A	3.039	0.712	1.908	2531	21
RL	4.965	1.245	2.780	3348	21
AL	6.358	0.521	5.940	5698	21
LM	15.940	1.748	14.581	14095	21
LI	5.575	0.440	5.458	4814	21
POR	8.830	0.787	8.573	7470	21
PL	7.401	0.865	6.389	5898	21

Table S4 (related to Figure 5): Measure of topography across areas. Percentage of voxels per area with significant retinotopic projection selectivity for V1.

		azimuth		elevation	
**Area**	**Voxel count**	**p<0.1 (%)**	**p<0.05 (%)**	**p<0.1 (%)**	**p<0.05 (%)**
AUD	1904	9.30	4.31	6.41	2.15
MO	738	7.45	1.90	12.06	2.57
SS	1456	9.00	1.17	14.42	1.58
ACA	870	6.44	1.03	9.89	2.64
CLA	242	13.22	2.48	6.20	3.72
TEa	1004	10.96	3.09	10.26	4.78
RSP	3262	16.62	4.69	12.05	3.83
ECT	281	6.41	1.07	7.83	0.36
SUBcom	292	13.70	2.40	5.82	1.71
LEC	335	5.97	0.90	1.19	0.60
MEC	556	4.68	0.90	3.78	0.90
PM	633	17.22	6.48	12.01	5.85
AM	453	13.47	7.51	10.82	7.06
A	595	13.95	2.86	10.59	7.23
RL	476	17.44	2.31	8.40	3.57
AL	463	14.25	6.91	13.17	7.78
LM	772	11.40	3.50	14.25	5.96
LI	335	10.15	3.88	10.15	4.18
POR	555	14.23	9.73	11.71	6.85
PL	399	11.78	5.76	10.53	6.27

Table S5 (related to Figure 6): Measure of bias in projections to V1. Correlation coefficient and p-values for cell count to azimuth and elevation retinotopy correlation. (p<0.05 in red)

Areas	Azimuth correlation r (p)	Elevation correlation r (p)
AUD	0.0188 (0.9448)	-0.3029 (0.2541)
MO	0.1845 (0.4939)	-0.0051 (0.9850)
SS	-0.0750 (0.7826)	0.1935 (0.4727)
ACA	0.1840 (0.4952)	-0.2517 (0.3470)
CLA	0.3484 (0.2031)	-0.2796 (0.3128)
Tea	-0.0101 (0.9705)	-0.4088 (0.1159)
RSPagl	0.7372 (0.0011)	-0.4618 (0.0717)
RSPd	0.5132 (0.0420)	-0.2747 (0.3032)
RSPv	-0.0275 (0.9196)	-0.2157 (0.4223)
ECT	0.4262 (0.0997)	-0.6089 (0.0123)
PRE	-0.1667 (0.5373)	0.4901 (0.0539)
POST	0.5202 (0.0389)	-0.1317 (0.6268)
PAR	-0.0269 (0.9242)	-0.3958 (0.1442)
LEC	0.0405 (0.8860)	0.0260 (0.9266)
MEC	-0.0076 (0.9785)	-0.0858 (0.7610)
PM	0.7284 (0.0002)	-0.6241 (0.0025)
AM	0.5138 (0.0172)	-0.3305 (0.1434)
A	0.3687 (0.1001)	-0.0384 (0.8687)
RL	-0.5545 (0.0091)	0.3757 (0.0933)
AL	-0.1493 (0.5183)	-0.0395 (0.8649)
LM	-0.3024 (0.1827)	0.4734 (0.0302)
LI	0.5161 (0.0166)	-0.5227 (0.0151)
POR	-0.4690 (0.0320)	-0.0161 (0.9448)
PL	-0.4902 (0.0241)	0.2231 (0.3310)

Table S6 (related to Figure 6): Layer distribution of cells in the source area projecting to V1, measured as the percentage of supragranular labelled neurons (%SLN).

Higher Visual Areas		Non-Visual Areas	
	**%SLN**		**%SLN**
**RL**	9.60	**MEC**	0.06
**POR**	10.00	**LEC**	3.51
**PL**	11.18	**ACA**	4.37
**A**	14.53	**MO**	6.42
**LI**	14.49	**RSPv**	8.36
**AM**	17.68	**TEa**	8.71
**AL**	18.74	**ECT**	10.02
**LM**	20.52	**SS**	12.78
**PM**	24.39	**AUD**	14.40
		**RSPd**	15.03
		**RSPagl**	22.74

Supplementary Figures

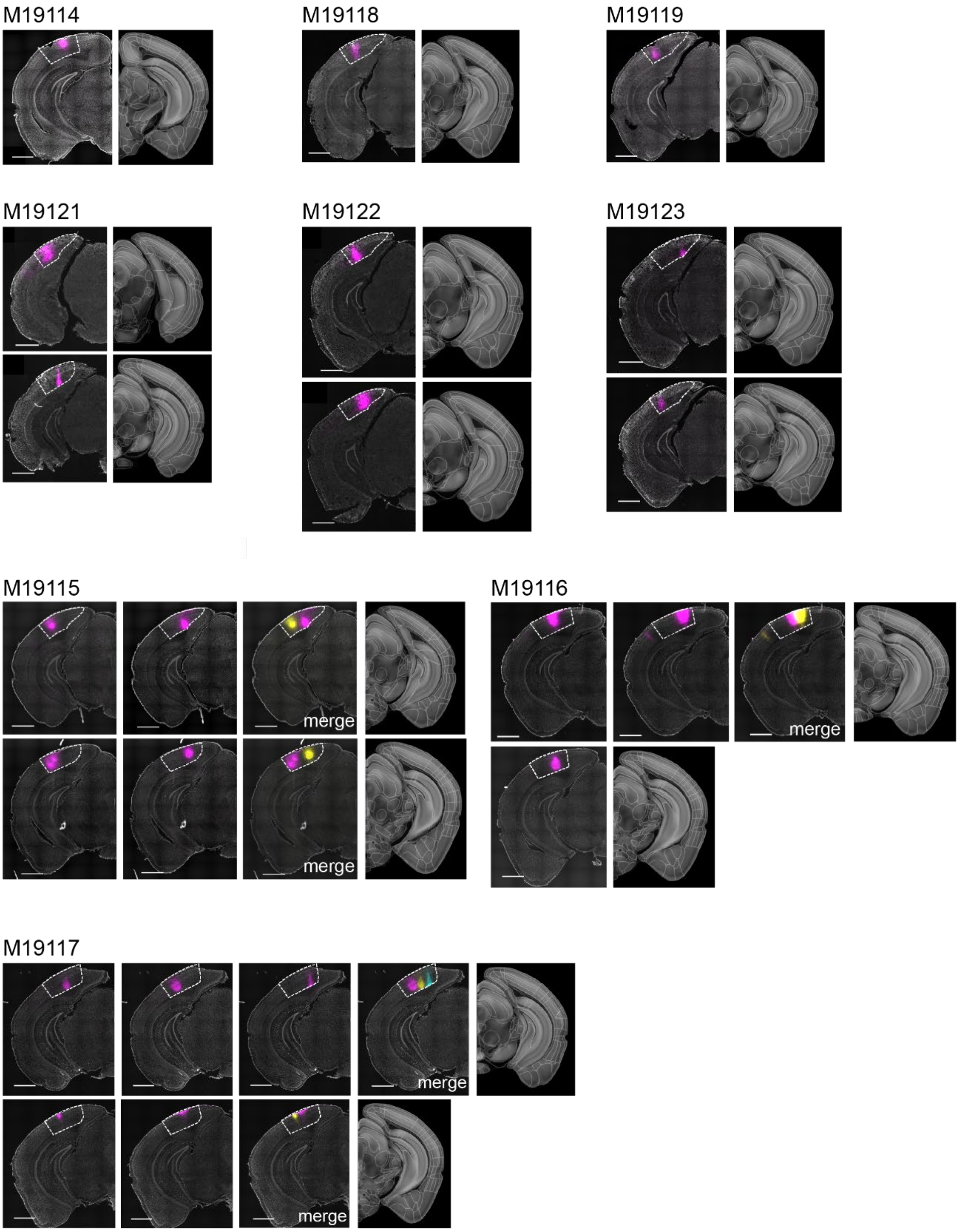

Figure S1 (related to Figure 1): CTB injection sites used in the study (9 animals, 21 injections). Fluorescent signal from each injection is shown in magenta. In 'merge' images for multiple injections within the same hemisphere, additional injections are shown in yellow or cyan. For each brain image (left hemisphere image), the ARA image used for alignment is shown on the same row (right hemisphere image). The brain images are pre-morphing, before alignment to the reference image. Border of V1 is indicated in white dotted lines according to this ARA image. Scale bars = 1mm. Further details of injections in methods and Table 1.

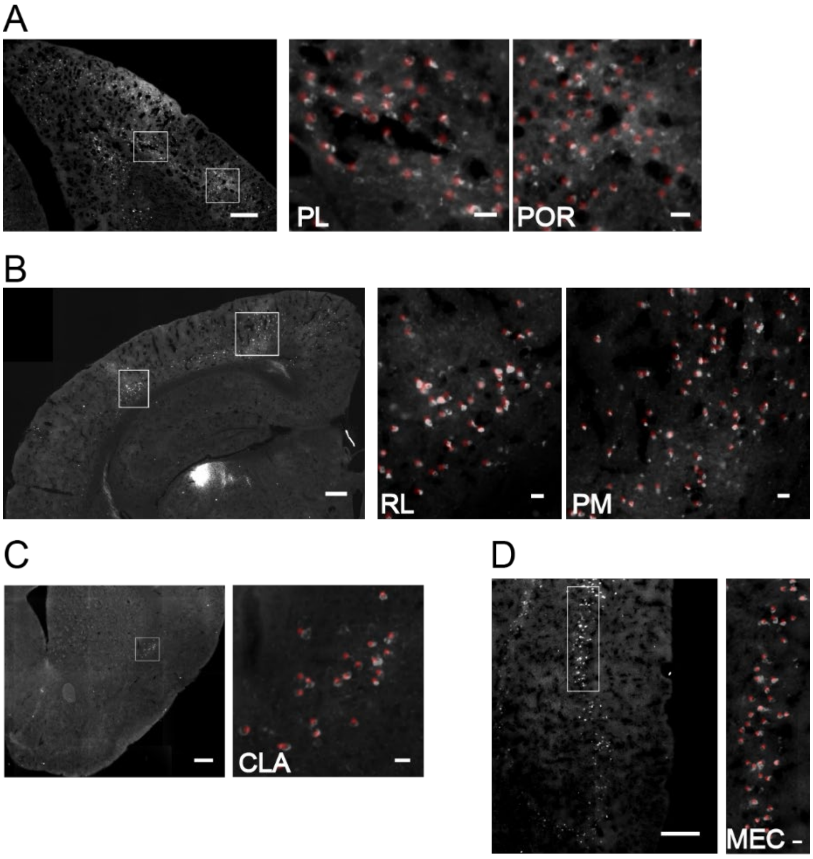

Figure S2 (related to Figure 1): Efficacy of cell-detection. **A-D)** Cell detection through our software pipeline. Red regions indicate detected cells. Right panels correspond to white boxed regions on the left panel. Scale bars: left panel = 100µm, right panel = 10µm.

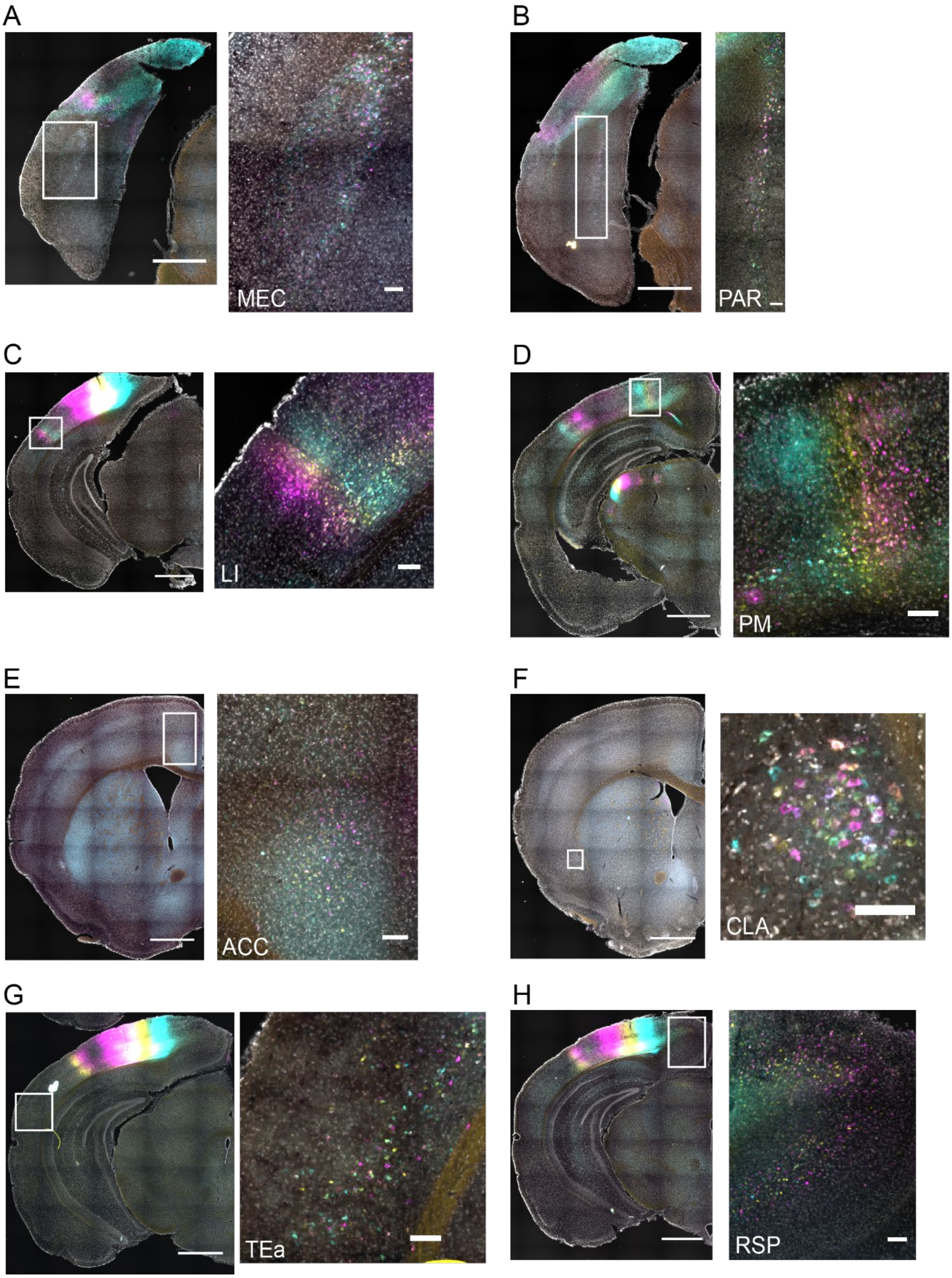

Figure S3 (related to Figure 2): Retrogradely labelled cells detected in various brain areas. **A-H)** Example areas showing retrogradely labelled cells. Right panels correspond to white boxed regions on the left panel. Scale bars: left panel = 1 mm, right panel = 100 µm.

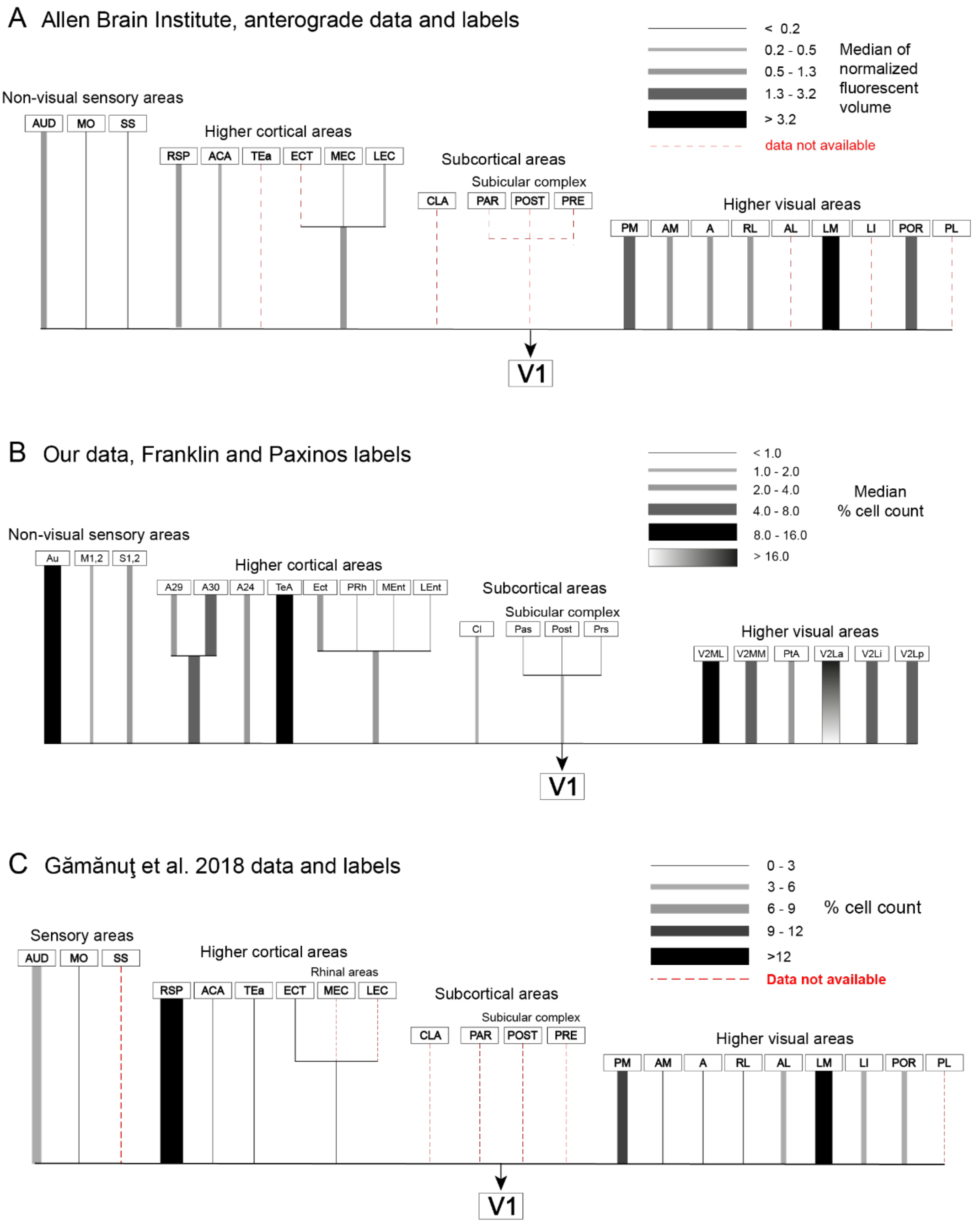

Figure S4 (related to Figure 3): Proportion of projections to V1 based on different atlases, and data. **A)** Estimated projection to V1 from brain wide anterograde injection data from the Allen Brain Institute. Injections to these areas were curated for specificity and projection to V1 from each injection was quantified (volume of fluorescent pixels in V1 normalized by injection volume). See methods for further details. **B)** Our retrograde data, recalculated with FP labels. **C)** Gamanut et al. retrograde data and labels. FP labels: Au: Auditory cortex; M1,2: Primary, Secondary Motor cortex; S1,2: Primary, Secondary Somatosensory cortex; A29, A30, A24: Cingulate cortex area 29,30,24; PRh: Perirhinal cortex; MEnt: Medial enthorhinal cortex; LEnt; Lateral enthorhinal cortex; Cl: Claustrum; Pas,Post,Prs: Para-, Post-, Pre-subiculum ; V2ML: Secondary visual cortex Mediolateral area; V2MM: Secondary visual cortex Mediomedial area; PtA: Parietal association cortex; V2La: Secondary visual cortex Lateralanterior area; V2Li: Secondary visual cortex lateralinferior area; V2Lp: Secondary visual cortex Lateroposterior area.

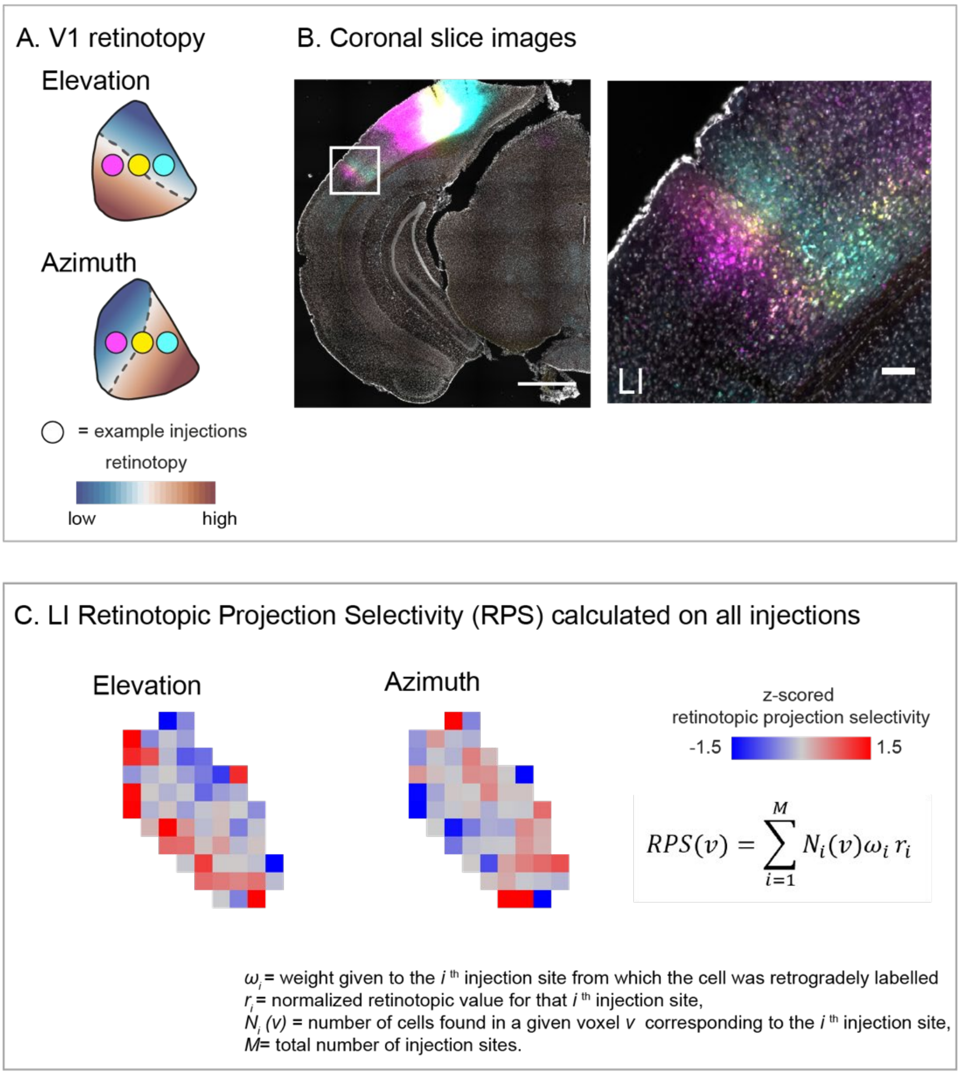

Figure S5 (related to Figure 5): Calculating retinotopic projection selectivity. A. The retinotopic map of V1 in elevation (top) and azimuth (bottom) with a schematic of the injection sites for the images in B, shown as coloured dots. **B.** Example coronal slice of animal with three injections showing retrogradely labelled cell. Right is a zoom into the area LI (same as Supplementary Figure 3C). Scale bars: left panel = 1 mm, right panel = 100 µm. **C**. Coronal projection of the retinotopic projection selectivity (RPS) for area LI calculated based on all the injections in our study. The pattern of mean RPS along the coronal slice shown in C is consistent with the multi-injection single slice image example shown in B. The expected retinotopic order of magenta, yellow and cyan cells are high to low in elevation, and low to high in azimuth (see A). This is captured by the ordering of mean RPS values in the coronal section (left to right edge).

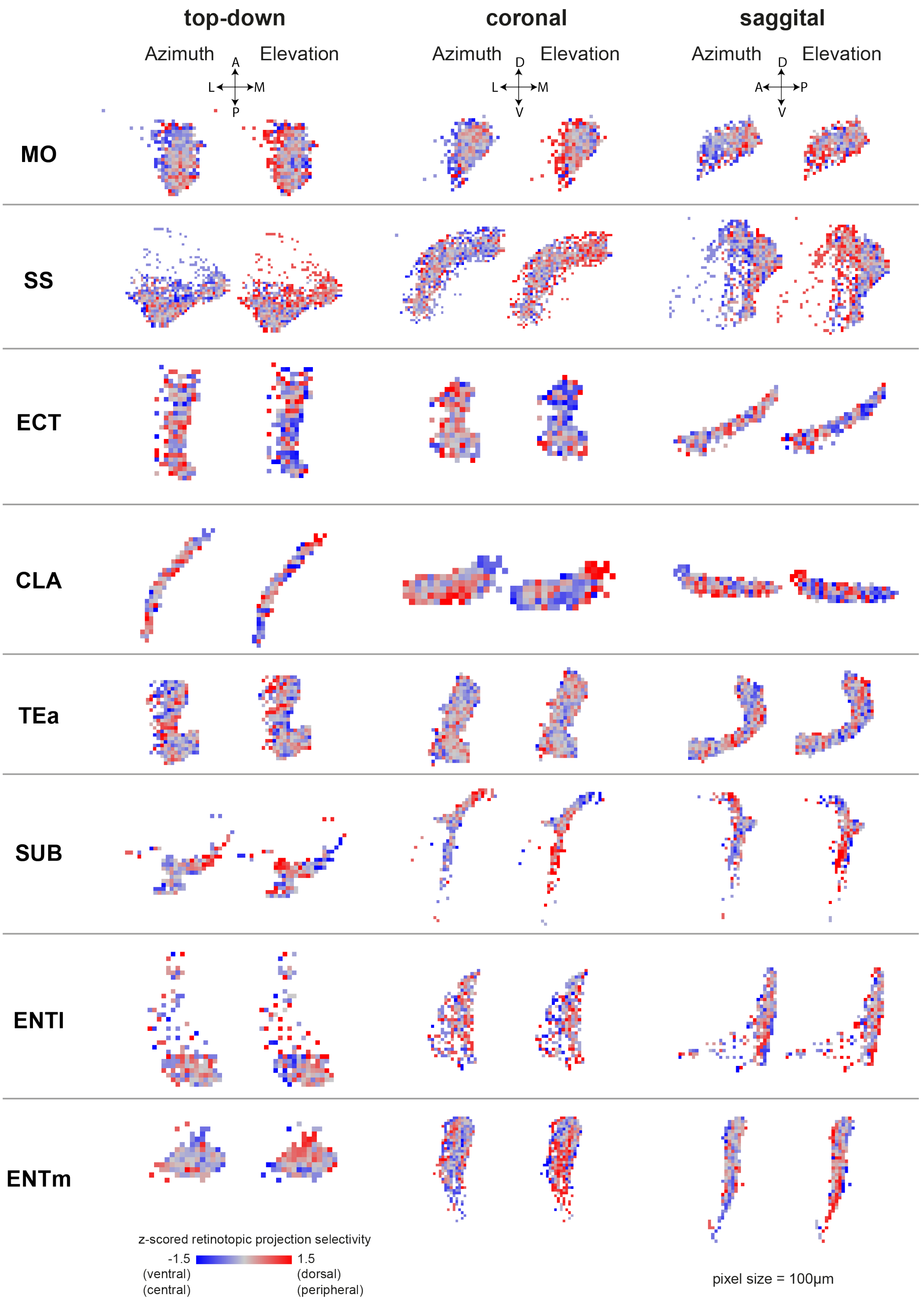

Figure S6 (related to Figure 5): Organization of cell bodies in non-visual areas projecting to V1. Each image represents one of the three views (top-down, coronal or saggital) of the 3D volume of each area. Normalized (z-scored) retinotopic projection selectivity, averaged across the top-down (left), coronal (middle) and sagittal (right) axes (binned in 100µm voxels and shown in the left hemisphere). Some non-visual areas are shown in Fig 5. Voxels are colour-coded based on their mean normalized selectivity along azimuth (left) or elevation (right) retinotopy. Only voxels that contained labelled cells are shown, causing some areas to have a sparse representation (for example SUB and ENTl).

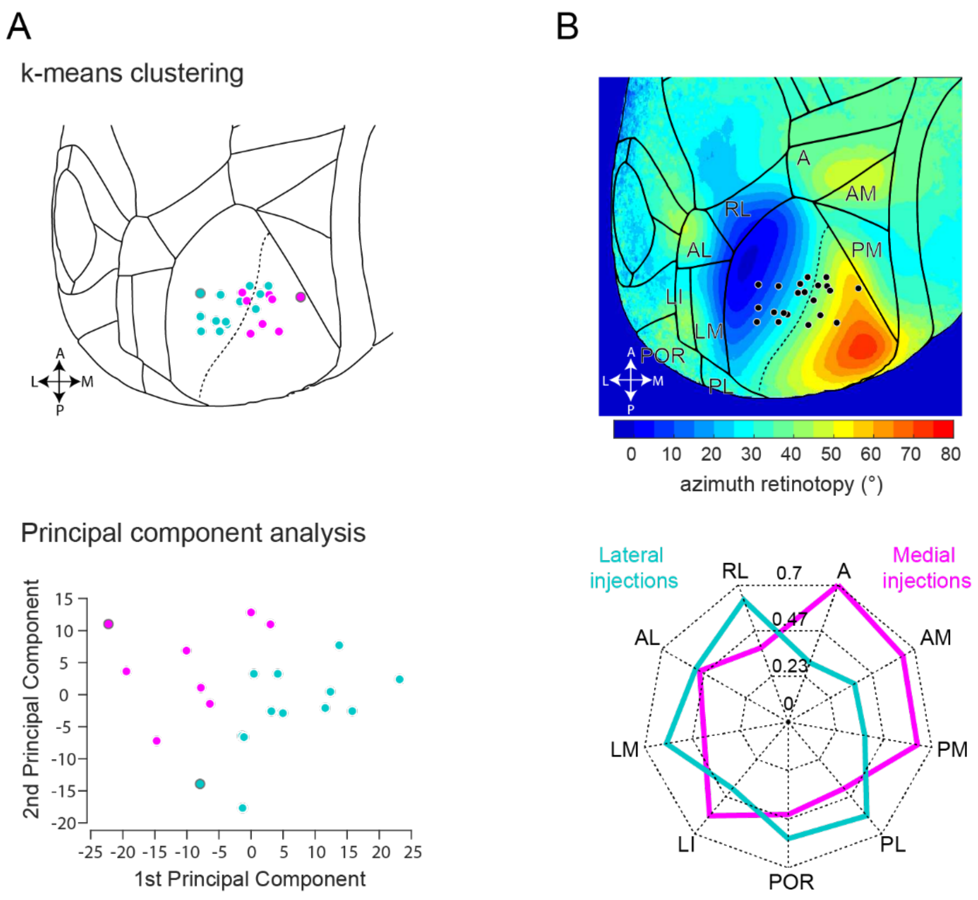

Figure S7 (related to Figure 7): A) Injection site clustering analysis: Top) k-means clustering resulted in two groups corresponding to medial and lateral portions of V1. Points with gray outline denote data points used as seeds (21 injections; 9 animals). Bottom) Applying this grouping in principal component space showed separation into two continuous groups. **B)** Injection site grouping analysis: Top) Injection coordinates plotted on mean azimuthal retinotopic map from Allen Institute. Contours are spaced 5° apart from −5° to 80° in azimuth. Bottom) Grouping injections into medial and lateral groups show higher cell counts in lateral HVAs for lateral injections, and in medial HVAs for medial injections.
